# Combined protein construct and synthetic gene engineering for heterologous protein expression and crystallization using Gene Composer

**DOI:** 10.1186/1472-6750-9-37

**Published:** 2009-04-21

**Authors:** Amy Raymond, Scott Lovell, Don Lorimer, John Walchli, Mark Mixon, Ellen Wallace, Kaitlin Thompkins, Kimberly Archer, Alex Burgin, Lance Stewart

**Affiliations:** 1 deCODE biostructures Inc, 7869 NE Day Road West, Bainbridge Island, WA 98110, USA; 2 Seattle Structural Genomics, Center for Infectious Disease, Bainbridge Island, WA 98110, USA; 3 Accelerated Technologies, Center for Gene to 3D Structure, Bainbridge Island, WA 98110, USA; 4 Emerald BioSystems Inc, 7869 NE Day Road West, Bainbridge Island, WA 98110, USA

## Abstract

**Background:**

With the goal of improving yield and success rates of heterologous protein production for structural studies we have developed the database and algorithm software package Gene Composer. This freely available electronic tool facilitates the information-rich design of protein constructs and their engineered synthetic gene sequences, as detailed in the accompanying manuscript.

**Results:**

In this report, we compare heterologous protein expression levels from native sequences to that of codon engineered synthetic gene constructs designed by Gene Composer. A test set of proteins including a human kinase (P38α), viral polymerase (HCV NS5B), and bacterial structural protein (FtsZ) were expressed in both *E. coli *and a cell-free wheat germ translation system. We also compare the protein expression levels in *E. coli *for a set of 11 different proteins with greatly varied G:C content and codon bias.

**Conclusion:**

The results consistently demonstrate that protein yields from codon engineered Gene Composer designs are as good as or better than those achieved from the synonymous native genes. Moreover, structure guided N- and C-terminal deletion constructs designed with the aid of Gene Composer can lead to greater success in gene to structure work as exemplified by the X-ray crystallographic structure determination of FtsZ from *Bacillus subtilis*. These results validate the Gene Composer algorithms, and suggest that using a combination of synthetic gene and protein construct engineering tools can improve the economics of gene to structure research.

## Background

The gene to structure endeavor faces intrinsic challenges at several steps in the experimental pathway. The primary challenge is expressing the great quantities of protein required to support structural studies, and this challenge can be magnified by the use and necessity of heterologous expression systems. While many research pursuits begin with protein purification, the extent of material required is a burden somewhat unique to protein crystallography. A second major problem of protein crystallography is identifying a suitable protein construct, given that small changes in the protein can have profound effects on crystallization [1,2]. These two problems are related since small changes in the protein construct can also have profound effects on the level of protein expression, solubility, and diffraction quality of protein crystals obtained. One recent report notes that construct engineering can double the number of targets expressing soluble protein in a heterologous system, and can provide a fourfold increase in the likelihood of obtaining well-diffracting crystals [2]. These aggregate improvements can enable extensive crystallization trials.

It is well known that codon utilization is highly biased and varies considerably among various organisms and codon usage is considered a key determinant of eventual heterologous protein expression [3]. Codon bias in genomes can be defined as the unequal usage of synonymous codons in known or predicted open reading frames (ORFs). Codon-usage patterns are related to the relative abundance of tRNA isoacceptors, and genes encoding highly expressed proteins show differences in their codon usage frequencies [4]. Extensive research by Karlin and Sharp have demonstrated that highly expressed genes in *E. coli *and other bacteria have a significant bias towards certain subsets of codons [5,6]. Moreover, bacteria and unicellular eukaryotic organisms seem to have codon biases that are highly correlated with measured isoaccepting tRNA levels [7-9]. In general, where it has been measured, higher eukaryotes also appear to have codon biases that complement the abundance of isoaccepting tRNAs [10,11].

It is generally thought that codon usage alters peptide elongation rates, however codon-usage patterns can also improve the fidelity and kinetic efficiency of translation [12]. Since the level of isoaccepting tRNAs in *E. coli *are correlated with codon bias, it is likely that the presence of rare codons has a negative impact on expression of heterologous genes. It is also likely that the kinetics of translation are improved by more closely matching the codon usage of a recombinant gene product to the usage of the expression host [3,13-17]. Apart from the nonrandom use of codons, it has also been noted that codon/anticodon recognition is influenced by sequences outside the codon itself, a phenomenon termed codon context [18-20]. There is, for example, an occurrence bias between specific adjacent codon pairs and these biases are different for highly expressed versus low expressed proteins in *E. coli *[21-23]. Clearly, there are still a number of mysteries in the subject of codon bias and codon context. Taken together these results also emphasize how difficult it is to create "codon optimized" genes for expressing proteins since the rules are complex and not yet fully defined.

As noted recently, more empirical evidence about protein expression from engineered genes is still needed, and efforts are being made to database the outcomes of gene engineering [24,25]. There remain a variety of other factors that should be considered when engineering synthetic genes and the details of "optimal" gene design continue to evolve [26]. For example, the synthetic gene sequence should not only contain the proper codon bias and codon context, but should also not result in mRNA secondary structures that may inhibit translation [27]. Additional considerations such as unusual sequence repeats, and cryptic regulatory sequences (*e.g*. Shine-Dalgarno, splice sites, RNase cleavage sites, *etc.*) can impact the fidelity and yield of protein expression [28].

Large scale projects in genomic sequencing and protein structure determination are producing enormous quantities of data on the relationships between 2D gene sequence and 3D protein structure. Moreover, such efforts are providing experimental data on success factors at every step in the gene to structure research endeavor. This wealth of information should be used in a feedback cycle to facilitate the design and production of genes and protein constructs that are engineered for the successful production of functional protein samples for structural studies. Fundamentally, this goal represents a bioinformatics software challenge.

To better address this issue in the context of synthetic gene design, we have created a Protein Construct Design interface for Gene Composer which distills protein structure information from PDB files and comparative sequence information into a graphical user interface that allows the user to simultaneously analyze known protein structures, together with homologous target protein sequences for which derivative constructs can be designed. This interface allows the user to simultaneously understand sequence conservation, ligand contacts, and known or predicted structural elements to define candidate protein constructs for crystallization and then design harmonized nucleic acid sequences to express those proteins.

To demonstrate the utility of this approach, we have used Gene Composer to design protein constructs, as well as the synthetic gene sequences to express those proteins. We compared the resulting protein expression level to full length proteins expressed from native genes. We initially chose to examine proteins from three different sources (human, viral and eubacterial) in two different heterologous expression systems (*E. coli *and wheat germ *in vitro *cell free expression). The results show that Gene Composer designed proteins and their corresponding engineered genes can be expressed at significantly higher levels than native genes. In addition, the results show that using Gene Composer to design protein constructs can significantly improve the likelihood of obtaining protein crystals.

## Results and discussion

### Construct Design Using Gene Composer™ Software

**Figure 1:**
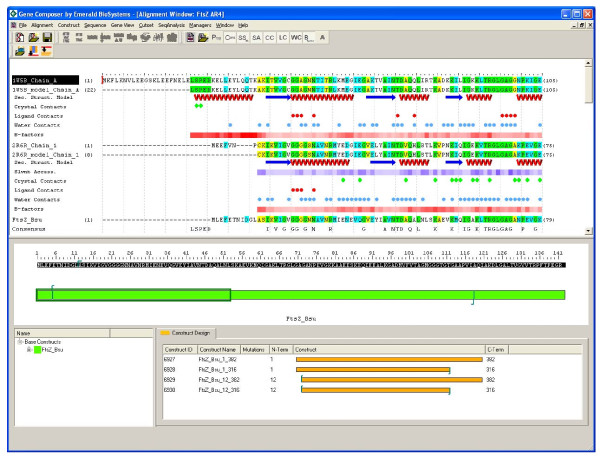
**Gene Composer software**. Comparative sequence alignments and corresponding structural information are organized by the Alignment Viewer of Gene Composer (top portion of panel). Amino acid sequence of *Aquifex aeolicus *FtsZ used to obtain FtsZ crystal structure (2R6R) is shown; Chain sequence indicates amino acid residues present in the protein constructs and Model sequence indicates amino acid residues that could be modeled from the electron density maps. Protein secondary structural regions are represented by red helices for alpha helical regions, blue arrows for beta-sheet regions, and green squiggles for turns, as defined the respective PDB file format. Thermal B-factors from the PDB file are normalized and represented in red shading levels (light is low and dark is high). The crystal, ligand, and water contacts are all extracted from the PDB file and listed pictorially. The *bacillus subtilis *FtsZ sequence (target) is shown on the bottom line and is aligned with FtsZ sequences from multiple species (accession codes indicated). Conserved residues are highlighted (green = high; blue = medium; yellow = low), and a consensus sequence is listed in the bottom row of the alignment.

In order to compare protein expression from engineered genes vs. native genes we chose to examine genes with significantly different G:C content and codon usage bias. We examined three globular proteins from three phylogenetically disparate sources including: FtsZ, the cell division protein from *B. subtilis*; NS5B, the RNA-dependent RNA polymerase from Hepatitis C Virus; and P38α, a human protein kinase. Using Gene Composer software, we made an alignment of each protein with published structural information, as well as the sequences of several homologous proteins for which structural information is not available. These alignments provided insights into the conservation of amino acid sequences and the known structure of homologous proteins that can be combined to engineer superior protein constructs for expression testing and crystallization trials. As an example, The Protein Alignment viewer of FtsZ (Figure 1) shows that the first eleven N-terminal amino acids of *B. subtilis *FtsZ are not conserved among several closely related species (*Clostridium sp*., *Listeria sp*., *Staphylococcus sp*., and *Streptomyces sp*.). In addition, the homologous N-terminal region of *Aquifex *FtsZ (PDB ID 2R6R) is present but disordered in the crystal lattice (compare 2R6R chain sequence to 2R6R model sequence). Together, this information suggests that this N-terminal region is not important for the function and structure of *B. subtilis *FtsZ, and that removal of this region could improve protein expression. A similar analysis of the C-terminal region of FtsZ showed that a C-terminal region (Glu317-Gly382 in *B. subtilis *FtsZ) was not conserved and not present in several other constructs used to generate FtsZ crystals (including 2R6R). Based on this analysis we defined a truncated form of *B. subtilis *FtsZ spanning residues Ala12-Ile316 (termed "FtsZ truncated").

A similar analysis was performed with NS5B and showed that C-terminal residues were not conserved and not visible within the electron density maps of several crystal structures. From this analysis, we identified a C-terminal truncation spanning residues 1–568 for expression testing. Finally, an analysis of P38α showed that residues across the entire protein were relatively highly conserved and almost all residues could be visualized in published crystal structures. Based on these results, we did not create a truncated form and only attempted expression of the full-length protein.

At this point, each target was moved into the Gene Design module (Protein-to-DNA) of Gene Composer™. For engineered genes, the nucleic acid sequence was determined by back-translation from the amino acid sequence. As previously described [29], multiple factors were incorporated in the final design including *E. coli *codon usage, overall G:C content, potential mRNA secondary structures, presence of out-of-frame stop codons, removal of cryptic Shine-Dalgarno sequences, removal of repeated strings of the same nucleic acid sequence, as well as the silent introduction and removal of select restriction sites to facilitate cloning. For native genes, the wild-type sequences were input with no sequence alteration. The resulting full length engineered genes and native genes express identical proteins, and were cloned into identical expression vectors.

### Protein Expression in *E. coli*

In order to determine if the use of phylogenetic and structural information to design modified protein constructs and engineering of the nucleic acid sequence resulted in improved protein expression, we compared the level of protein expressed from the engineered synthetic genes to proteins (constructs listed in Table 1) expressed from native sequences under identical conditions. It has been well demonstrated that expression conditions (media, temperature, inducer concentration, length of induction, etc.) can have a profound effect of the level of protein expression and it is certainly possible that some or all of these variables could be optimized to alter the relative expression levels between the engineered proteins; however we first chose to compare the expression under a single condition. This condition was based on an analysis of expression conditions used by multiple high throughput protein structure initiative centers and we believe a typical representative of a common "first pass" expression condition (0.4 mM IPTG inducer at mid-log phase at 30°C for four hours in 2YT media).

**Table 1:** Protein constructs used in this test set.

**Target**	**Construct**	**aa**	**MW (kDa)**
**FtsZ**	Full Length	1–382	42.8

	truncated	12–316	34

			

**NS5B**	Full Length	1–589	66.4

	truncated	1–568	64

			

**P38α**	Full Length	1–370	43.5

**Figure 2:**
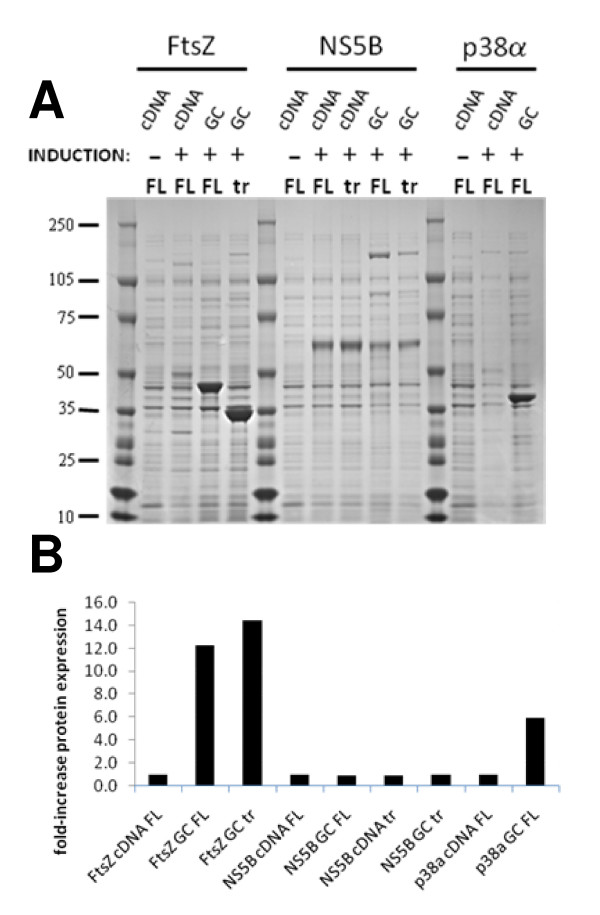
**Expression of full length and truncated native and Gene Composer engineered genes in *E. coli***. **A**, Total soluble protein was resolved by SDS-PAGE and the resulting commassie blue stained gel is shown. Lanes containing *B. subtilis *FtsZ (full length: 42.8 kDa, truncated: 34.0 kDa), Hepatitis C Virus NS5B (full length: 66.4 kDa, truncated: 64.0 kDa); and human P38α (full length: 43.5 kDa) are indicated. Lanes from Gene Composer engineered genes are indicated (Eng) and compared to native genes (Native). Expression of full length (FL) and truncated forms (tr) proteins is also compared. Negative control experiments showing expression in the absence of inducer (-) is also shown. **B**, For each sample, the amount of protein observed was divided by the amount of full length native protein and plotted. By definition, FtsZ native FL, NS5B native FL, and P38α native samples have values of 1 and are shown as controls.

The results in Figure 2 show that the amount of soluble protein produced by the Gene Composer engineered genes for all three targets are either equal to or greatly improved over the level of protein expressed from the native gene. This improvement was most dramatic in the case of FtsZ where gene engineering resulted in a 12-fold increase in the amount of soluble protein from the same weight of cell paste (full-length native vs. full-length engineered). The deletion of N-terminal and C-terminal residues did not significantly improve expression relative to the full length FtsZ protein (15-fold increase relative to expression from full-length native gene). The expression of P38α from the synthetic gene was also significantly improved (6-fold) relative to expression from the wild-type P38α gene. It is important to emphasize that synthetic gene engineering using Gene Composer does not always result in improved protein expression, and there was no clear improvement of expression of NS5B compared to native NS5B. We also compared the expression of four different human GPCR membrane proteins from engineered and native genes: human adenosine A2a receptor (Adora2a), human adrenergic, beta-2 receptor (b2AR), human tachykinin receptor 1 isoform long (NK1), human Galanin receptor type 1 (GalRI). These GPCRs were expressed as N-terminal Mistic fusions [30,31], and in all cases the level of expression from the engineered gene was the same or slightly higher when compared to expression from the native gene (data not shown).

### Crystallography and Structure Determinations

**Figure 3:**
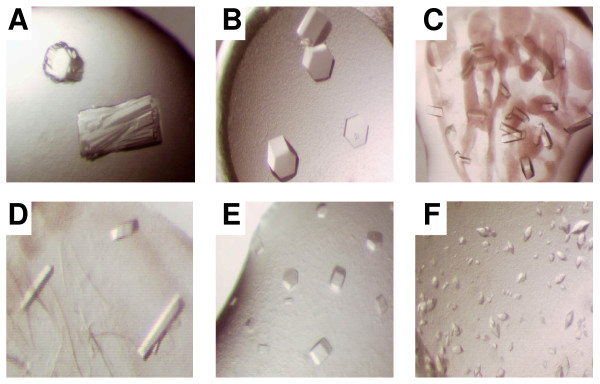
**Crystallization of FtsZ**. Protein crystals of FtsZ obtained under different condtions are shown: **A**, 1.26 M (NH_4_)_2_SO_4_, 100 mM MES pH 6.5. **B**, 1.26 M (NH_4_)_2_SO_4_, 100 mM Hepes pH 7.5. **C**, 30% (v/v) PEG-200, 100 mM MES pH 6.0, 5% (w/v) PEG-3000. **D**, 20% (w/v) PEG-8000, 100 mM Hepes pH 7.5. **E**, 1.26 M (NH_4_)_2_SO_4_, 100 mM Hepes pH 7.5. **F**, 50% (v/v) PEG-200, 100 mM Tris pH 7.0, 0.05 M Li_2_SO_4_.

In order to determine if proteins expressed from the synthetic genes could be used successfully in protein crystallization experiments, we scaled up the expression and purified full length and truncated forms of FtsZ. The purified proteins were concentrated and used in a series of random sparse matrix crystallization screens. For the full-length FtsZ protein, we established 768 random sparse matrix sitting drop crystallization trials, but did not observe any crystallization hits after monitoring trials for one month. 192 different random sparse matrix crystallization trials were established with the truncated construct (FtsZ truncated) and 32 different crystallization hits were observed following 1 week of monitoring. Representative examples of these crystal hits are shown in Figure 3. Based on the quality of crystal morphology and initial diffraction screening experiments, we focused on a single condition (30% PEG-200, 100 mM MES pH 6.0, 5% PEG-3000) and harvested crystals that diffracted X-rays to 2.45Å resolution. The structure [PDB code: 2RHL] was solved by molecular replacement as described in the Materials and Methods.

**Figure 4:**
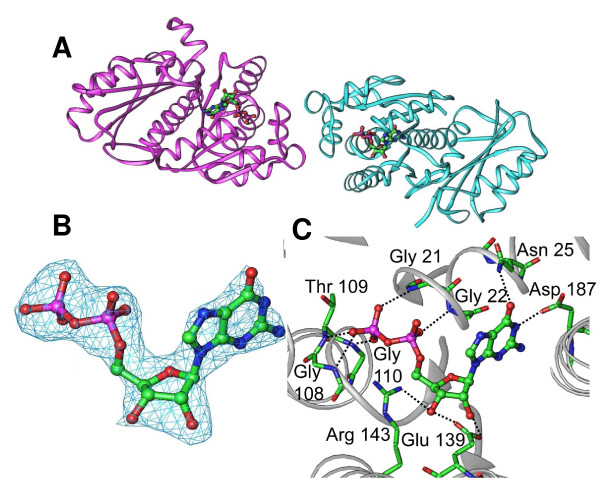
**Structure of *Bacillus subtillis FtsZ***. **A**, Ribbon diagram of the non-crystallographic dimers viewed approximately parallel to the NCS 2-fold axis is shown. Bound GDP within the active site is represented in stick drawing. **B**, The F_o_-F_c _omit electron density map contoured at 3σ is shown in blue. Stick representation of bound GDP is modeled in the omit density. **C**, Hydrogen bond interactions between FtsZ active site residues (stick) and GDP (ball and sticks) are shown as black dashed lines. Amino acids residues are numbered and the FtsZ backbone is shown in grey.

FtsZ formed orthorhombic crystals and contained two molecules in the asymmetric unit related by a non-crystallographic 2-fold axis (Fig. 4A). Non-bonded contacts at the NCS dimer interface are formed between the backbone oxygen of Lys 64 and the side chain of Asn 74 as well as Glu 76 and Glu 84. The latter are formed via water mediated contacts. The structure was traced from Ala 12 to Phe 315 of FtsZ for both subunits. Although guanosine diphosphate (GDP) was not added during the purification or crystallization, analysis of the nucleotide binding pocket following refinement revealed prominent difference density (Fig. 4B) consistent with the presence of GDP. The presence of GDP in the active site of FtsZ has been observed before [32,33] and suggests that the crystallized protein is functional. GDP forms direct non-bonded contacts with the T1 loop (Gly 21-Gly 22) and T4 loops (Gly 108-Gly 110) (Fig. 4C). The latter corresponds to the tubulin signature motif (GGGTGTG) which is involved in phosphate binding [34]. These domains are in similar orientations to domains previously described [35,36].

It has been shown previously that nucleotide exchange occurs readily and has negligible conformational effects in certain FtsZ structures[33]. To determine if this was the case for the *B. subtilis *FtsZ structure, crystals of FtsZ:GDP were soaked in the presence of GTP-γ-S. The crystals (FtsZ:GSP) tolerated the soaks well and diffracted to the same resolution (2.45Ǻ) as FtsZ:GDP crystals. Similar to FtsZ:GDP, this structure [PDB code: 2RHO] was traced from Ala12 to Phe315. However, the residual electron density at the C-terminus of subunit B was weak and the purification tag could only be traced to Leu316. Also, residues Lys221 and Gly222 on chain B were disordered and were not modeled. Examination of the difference electron density revealed that subunit A contained GTP-γ-S as there was clear density for the γ-phosphate. However, the GDP in subunit B was not exchanged as evident by difference density which was consistent with GDP. Interactions with GTP-γ-S and FtsZ are similar to those observed in the FtsZ:GDP structure except that the γ-phosphate forms contacts with Ala71 and Ala73 of the T3 loop.

Two additional crystal forms were obtained in the presence of lithium sulfate. A primitive tetragonal form, FtsZ:SO_4_, was found to contain a single sulfate bound in the nucleotide pocket [PDB code: 2RHH]. This sulfate ion occupies position similar to that of the β-phosphate of GDP and GTP-γ-S observed for FtsZ:GDP and FtsZ:GSP. Not surprisingly, the sulfate ion forms non-bonded contacts with residues in the T1 and T4 loops. The sulfate ion is positioned in a nearly identical manner as that observed in a recently published full length *Bacillus *FtsZ structure [37]. A *C*-centered orthorhombic crystal form (FtsZ:2SO_4_) was found to contain two sulfate ions in the nucleotide binding pocket [PDB code: 2RHJ]. Additionally, prominent difference density was observed between the two sulfate ions which we clearly observed at a 6σ contour level. Initially, a water molecule was placed at this site however this water was surrounded by a large amount of positive difference electron density following refinement. The unidentified atom appeared to be coordinated by two water molecules and two oxygen atoms of the sulfates in a distorted square planar arrangement. We considered the possibility that this was a magnesium ion. However, although four coordinate magnesium is a possibility it typically forms six coordinate interactions[38]. We then used the program Wasp[39] to examine the solvent structure for potential metal ions. The water positioned at this site of positive difference density was flagged as a possible sodium ion. Therefore, a sodium ion was subsequently positioned in the difference density and refined. The refined metal-oxygen non-bonded distances and coordination geometry were consistent with those expected for a sodium ion[40]. In this structure, the sulfate ions adopt similar positions as the α and γ phosphates of GTP-γ-S found for FtsZ:GSP. Similarly, non-bonded contacts are formed between the T1, T3, and T4 loops of FtsZ and the sulfate ions.

These results clearly show that proteins expressed from a synthetic Gene Composer engineered gene can be successfully used in protein crystallization experiments. More importantly, the results also show that protein construct engineering using structural and phylogenetic information can be critical for successful crystallization of a protein target since only the truncated form of FtsZ yielded protein crystals.

### Protein Expression in *E. Coli *Under High-Throughput Conditions

In order to begin to understand how often engineering of the nucleic acid sequence will improve protein expression, we randomly chose eleven different proteins from three different bacterial species on the National Institute of Allergy and Infectious Disease priority pathogens list, and compared protein expression from full length wild type genes and full length Gene Composer engineered genes. The proteins were expressed from identical expression vectors, were amended with identical purification tags, and the final protein product pairs had identical amino acid sequences. All of the expressed proteins contained 6× His tags to facilitate quantitation of soluble protein expression. In these examples the proteins were expressed using autoinduction media [41].

**Figure 5:**
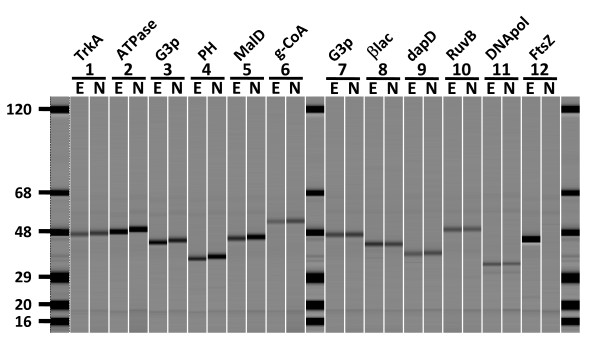
**Comparative analysis of protein expression from full length synthetic genes**. Proteins were expressed and purified in parallel from Gene Composer engineered (E) or native (N) genes as described in Materials and Methods (see Additional File 1). Proteins from *Brucella melitensis*, str. biovar Abortus 2308 were compared in pairs: **Pair 1**) Lactate/malate dehydrogenase:L-lactate dehydrogenase:TrkA potassium uptake protein (44.8 kDa); **Pair 2**) CbxX/CfqX superfamily:Disease resistance protein:ATP/GTP-binding site motif A (P-loop):AAA ATPase:AAA ATPase, central region (49.4 kDa); **Pair 3**) Glyceraldehyde 3-phosphate dehydrogenase:TrkA potassium uptake protein:Glyceraldehyde-3-phosphate dehydrogenase, type I (47.4 kDa); **Pair 4**) 3' exoribonuclease:Ribonuclease PH (37.0 kDa). *Burkholderia pseudomallei*, str. 1710b: **Pair 5**) malate dehydrogenase (46.2 kDa); **Pair 6**) glutaryl-CoA dehydrogenase (54.3 kDa); **Pair 7**) glyceraldehyde-3-phosphate dehydrogenase, type I (47.3 kDa); **Pair 8**) beta-lactamase (44.0 kDa); *Rickettsia prowazekii*, str. Madrid E: **Pair 9**) 2,3,4,5-Tetrahydropyridine-2-Carboxylate N-Succinyltransferase (dapD) (41.3 kDa); **Pair 10**) Holliday junction DNA helicase RuvB (49.6 kDa); **Pair 11**) DNA polymerase III subunit epsilon (37.2 kDa). *Bacillus subtilis*, subsp. Subtilis, str. 168; **Pair 12) **FtsZ (full length, 42.8 kDa). Molecular weight markers are present in lanes 1, 14, 27.

The results in Figure 5 show that the level of soluble protein expression is nearly identical for the randomly selected eleven pairs of constructs (see Additional file 1). As a control, the engineered and native FtsZ genes that had previously demonstrated significant differences in the level of protein expression under standard expression testing (see Figure 2) were included in the experiment. A similar result was obtained with FtsZ (see pair 12) with the engineered gene expressing at significantly higher levels than the wild-type gene, however there was only modest differences between the level of expression from the engineered gene vs. the native genes for the randomly selected constructs. This result demonstrates that redesign of the nucleic acid sequence does not always result in improved levels of protein expression; however it also demonstrates that gene engineering does not lead to decreased levels of protein production.

### Protein Expression in CFS

**Figure 6:**
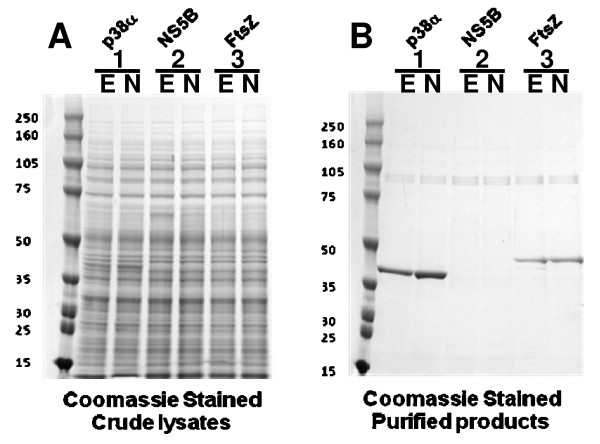
**Expression of full length native and Gene Composer engineered genes in Cell Free System, SDS-PAGE analyses**. **A**, Total protein from CFS expression of *B. subtilis *FtsZ (42.8 kDa), Hepatitis C Virus NS5B (66.4 kDa); and human P38α (43.5 kDa) were resolved by SDS PAGE and the resulting coomassie blue stained gel is shown. Odd lanes contain expression from native genes, even lanes contain expression from engineered genes. **B**, Soluble proteins from the total expression were purified by Ni affinity chromatography and resolved by SDS PAGE. Odd lanes contain expression from native genes, even lanes contain expression from engineered genes. Data shown is a representative from three independent experiments.

The improved expression of FtsZ and P38α from synthetic genes in *E. coli *may result from the biased codon usage of the native genes leading to abortive heterologous protein expression when rare tRNA pools become depleted *in vivo*. It is difficult to test this possibility *in vivo *because of the many factors that could potentially modulate levels of aminoacylated tRNA pools; however it is possible to control tRNA pools *in vitro*. To this end, we expressed several native and engineered gene sequences (optimized for *E. coli *expression) in a wheat germ extract-based Cell Free System (CFS, ). The cell free system reagents include an excess of molecular translation machinery, resulting in a protein expression strategy that is not limited by depletion of tRNA pools. We cloned the same engineered and native FtsZ, NS5B and P38α genes into a CFS expression vector (Materials and Methods) ensuring that the full length proteins had identical amino acid sequences. The proteins were expressed under identical conditions and all proteins present in the final expression extract (soluble and insoluble) were resolved by SDS PAGE. The resulting Commassie blue stained gel is shown in Figure 6A. The results show that because the recombinant expressed proteins FtsZ, NS5B and P38α only account for a small percentage of the total proteins present in the CFS extracts, an accurate comparison of expression levels of the proteins of interest cannot be made from the SDS-PAGE analysis of the total CFS extracts. Only the NS5B protein bands of ~ 60 KDa in lanes 3 and 4 of Figure 6B could be reliably discerned above the background proteins, and it appears that the native gene expressed NS5B protein in lane 3 is made in slightly larger amounts than its counterpart in lane 4 produced by the synthetic NS5B gene.

In order to more accurately compare the protein expression levels, the soluble 6X-His tagged recombinant proteins were purified from the CFS extracts by nickel chelate bead capture and resolved SDS PAGE with Commassie blue staining as shown in Figure 6B. The NS5B proteins were not captured by nickel chelate and were shown to be located entirely in the insoluble fraction of the expression extract (data not shown). Quantification of the full length purified FtsZ proteins shows an approximate 2-fold increase in protein expressed from the engineered gene relative to the native gene, while expression of full length P38α from both sources is equal. Thus, the difference in protein expression levels from synthetic engineered P38α and FtsZ genes relative their native counterparts is modest in the CFS experiment, and do not recapitulate the outcomes observed when these genes were expressed in *E. coli *cells (see Figure 2). Taken together, these results suggest that the rare codons present in the native gene sequences of human P38α and *B. subtilis *FtsZ can significantly hamper protein expression of these proteins in *E. coli*, but this can be overcome either by codon engineering for *E. coli *or by providing an excess of molecular translation machinery in the wheat germ CFS system. Moreover, the synthetic codon engineered versions of these genes do not appear to offer any significant advantage for protein production in the CFS wheat germ extract.

In order to explore the possibility that codon engineering of synthetic genes for high level *E. coli *expression could affect protein production in a wheat germ extract, we compared the CFS expression of synonymous pairs of genes from *Arabidopsis thaliana *whose codon usage is comparable to that of wheat, wherein the native *A. thaliana *gene is compared to that of a synthetic codon engineered gene designed with codon usage of wheat genes. In all cases, the level of CFS expression from the wheat codon engineered gene was the same or slightly higher than expression from the native *Arabidopsis *gene (data not shown). This data is consistent with the suggestion that translation machinery is not limiting for protein production in the CFS wheat germ extract.

## Conclusion

Obtaining sufficient quantities of soluble protein for structural studies often requires expressing proteins in heterologous expression systems. Unfortunately, the native nucleic sequence of any gene is likely to be biased from the evolution of that gene for expression in the native host, and in some cases, the gene may have selective pressures to be naturally expressed at very low levels. This bias may affect codon usage, codon context, mRNA secondary structures, regulatory elements, *etc*. which will likely impact the level of expression in a heterologous system. In order to overcome this fundamental problem, we have developed Gene Composer software to engineer any nucleic acid sequence for any given expression system (sister manuscript). In this report, we have systematically compared protein expression from native genes to protein expression from genes specifically engineered for high level expression in *E. coli*.

The majority of genes tested did not result in improved levels of protein expression. There are several possible explanations for this result. First, the yield of soluble protein may be dominated by the balance of protein turnover. If a protein is extremely unstable, increases in the rate of translation or fidelity of translation will have only small effects on the steady concentration of that protein in the cell. Future pulse-chase experiments designed to access rates of protein translation are necessary to address this possibility. Another possibility is that the results reflect our lack of understanding of the many factors that are important for protein expression and the likelihood that the "rules" are different for individual genes and proteins. For example, the engineered synthetic genes were harmonized for codon usage, absence of sequence repeats, absence of repeating codons within a sequence, reduction of local mRNA secondary structures within the transcript, and removal of cryptic Shine Dalgarno sequences; however, other factors may also be important for expression. It is not possible to test these unknown factors at this point; but synthetic gene design offers the ability to incorporate and test new gene design features as they become known.

Because we are still in the early days of discovering all of the rules that govern gene optimization, it is important to determine if Gene Composer designed genes reduce the level of protein expression compared to native genes. Engineered genes did not result in a decrease in the level of soluble protein from the 14 different genes tested, and in two cases, gene engineering resulted in a significant increase in soluble expression (FtsZ from *Bacillus subtilis *and human P38α). The underlying cause for the dramatic increased expression of FtsZ from the engineered synthetic gene is not immediately clear and likely results from a combination of factors. For example, comparison of expression of FtsZ in *E. coli *vs. cell free expression may suggest that codon usage resulted in a decrease in expression from the native gene since both engineered and native FtsZ genes expressed at relatively high levels in this heterologous system. The native FtsZ gene contained a total of 19 rare codons (1.58% of all codons) which were eliminated in the engineered gene (see Table 2), however NS5B contained a significantly larger number of rare codons (62 or 3.46% of all codons) but elimination of rare codons from NS5B had no effect on the level of expression. The total number of rare codons may not be important if the location of rare codons relative to individual folding protein domains or mRNA secondary structure elements could also affect protein expression. There are clearly a large number of different factors that could be the cause of the significant increase in FtsZ expression.

**Table 2:** Summary of rare codon usage in full length native genes.

**FtsZ FL native**		
**AA**	**codon**	**number (%)**

Arg	AGG	0

Arg	CGA	1 (6%)

Arg	CGG	0

Gly	GGA	12 (31%)

Gly	GGG	3 (8%)

Leu	CTA	2 (7%)

Pro	CCC	0

Ser	AGT	1 (6%)

		

**NS5B FL native**		

**AA**	**codon**	**number (%)**

Arg	AGG	8 (21%)

Arg	CGA	7 (18%)

Arg	CGG	7 (18%)

Gly	GGA	6 (17%)

Gly	GGG	11 (31%)

Leu	CTA	9 (14%)

Pro	CCC	13 (42%)

Ser	AGT	1 (2%)

		

**P38α FL native**		

**AA**	**codon**	**number (%)**

Arg	AGG	6 (30%)

Arg	CGA	1 (5%)

Arg	CGG	3 (15%)

Gly	GGA	4 (22%)

Gly	GGG	3 (17%)

Leu	CTA	3 (7%)

Pro	CCC	4 (22%)

Ser	AGT	1 (4%)

Codons rarely found in *E. coli *highly expressed genes are listed on the left. The number of occurrences of each rare codon in the wild type FtsZ, NS5B and P38α full length genes are listed. The percentage of occurrence (number of occurrences/total number of codons for each amino acid) is listed in parentheses.

The ability to express significant quantities of FtsZ enabled the crystallization of this target and demonstrates the utility of Gene Composer for protein design and construct engineering for structural studies. Specifically, we used Gene Composer to define structure-guided protein constructs for crystallization studies and showed that we were unable to obtain protein crystals of full length FtsZ, but did obtain multiple crystal hits from the truncated, engineered FtsZ construct (see Figures 1 and 3).

In conclusion, these results demonstrate the utility Gene Composer for the design of protein constructs and synthetic genes to express those proteins. The use of protein engineering to improve crystallization can have a significant impact on the gene to crystal structure process since very complex protein construct engineering principles (fusions, rearrangements, rationale surface variants, *etc.*) can be rapidly implemented without multiple molecular biology steps that can require significant amount of time and effort. Because engineering of the nucleic acid sequence did not decrease the level of protein expression and in some cases dramatically improved expression, synthetic gene design can also significantly improve the gene to crystal structure process. The utility of Gene Composer for engineering nucleic acid sequences will improve as more design principles are identified, especially as the cost of gene synthesis continues to decrease and robust protocols for rapidly synthesizing genes become more available.

## Methods

### Gene Composer engineering

Gene Composer engineering considerations included codon usage frequency minimum threshold of 2%, elimination of undesired restriction sites, elimination of five or more nucleotide repeats, placement of stop codons every 100 bp in second and third reading frames, elimination of cryptic Shine-Delgarno sequences, and optimized T_m _and ΔG. Detailed information about engineering with Gene Composer can be found in (Sister Manuscript).

### Cloning

All amplifications were performed in 50 μl reactions using KOD Hi-Fi polymerase (Novagen) according to the manufacturer's instructions. Resulting PCR products were resolved on 1% agarose gels in 1× TBE + EtBr, and subsequently gel-purified using Qiagen gel extraction purification protocol. Amplification products not requiring gel purification were purified using the microfuge PCR purification kit protocol. DNA yields were assessed with a Beckman Coulter DU 500 spectrophotometer. Ligation reactions were performed with T4 DNA Ligase (NEB), using a 1:3 molar ratio of vector and insert DNA. Chemically competent OneShot TOP10 cells were transformed with 6 μl of each ligation using a standard heat shock protocol. Cells were pelleted, resuspended in 50 μl, and spread on 10 cm 2XYT plates containing the appropriate antibiotic. Following 16 hour incubation at 37°C, transformant colonies were screened by PCR and positive clones were sequence verified.

#### Bacterial expression of P38α, NS5B, and FtsZ

Gene Composer engineered and native full length FtsZ genes were purchased from DNA2.0, and subcloned into pET28 using by AflIII/BamHI digestion. The engineered genes were optimized for expression in E. coli using E. coli codon usage tables and design algorithms (Sister Manuscript). For the eight other constructs in this test set, sequence verified, pCRBluntIITopo clones were amplified by PCR. Truncated FtsZ native and Gene Composer engineered inserts were amplified with primers to incorporate a 5' NcoI site before the first Met ATG, and adding two stops with a 3' BamHI site following the affinity tags. Native P38α was amplified using primers incorporating a flanking 5' NdeI site such that after subcloning, the 5' ATG would fall in-frame with the vector derived N-terminal tags. The 3' primer added two stop codons and a BamHI site. The resulting PCR product was purified using the Qiagen PCR purification kit, and sequentially digested by first NdeI (Fermentas), ethanol precipitated and digested with BamHI (Fermentas). Due to an internal NdeI site, two digestion fragments were gel-purified: a 5' 238 bp fragment, flanked on both sides by NdeI, and a 3' 857 bp fragment, with 5' NdeI and 3' BamHI termini. A 3-fold molar excess of pooled fragments was combined with NdeI/BamHI-digested pET15b in a standard ligation. Gene Composer engineered P38α was cloned in the same fashion, except that no internal NdeI site was present. The four NS5B constructs were amplified with primers which add a 5' NcoI site flanking the initiating methionine ATG, followed by a 6× His tag. Two stop codons were added after the last amino acid, followed by a 3' BamHI site for subcloning into NcoI/BamHI-digested pET15b. Cloning of full length and truncated wild-type NS5B was complicated by the presence of two internal NcoI restriction sites; desired constructs were the product of multiway ligation into pET15b. All transformant colonies were screened by PCR, and sequence verified.

#### Cell free expression of P38α, NS5B, and FtsZ

Sequence verified whole gene synthesis products of native and Gene Composer engineered versions of P38α and NS5B were used as PCR templates for cloning into vector pEU-E01. Full length native and Gene Composer engineered FtsZ ORFs were ordered from DNA 2.0, and served as template for cloning of the FtsZ expression constructs. Amplification primers for all six constructs added a 5' SpeI site and 3' XhoI site for cloning into SpeI/XhoI-cut pEU-E01 (CFS). Following spin purification of PCR products, approximately 1 ug PCR product was digested with SpeI/XhoI (Roche) and gel purified. Ligations were set up as described previously, and transformation reactions were spread on 2XYT ampicillin 100 ug/ml plates.

### Expression

#### *E. coli *expression of pET P38α, NS5B, and FtsZ constructs

Cultures of 2YT supplemented with antibiotic were inoculated with single colonies of BL21(DE3) cells that had been transformed with each sequence verified pET28 or pET15 construct. Following overnight growth at 37°C, cultures were diluted 100-fold in the same medium to a final volume of 1L, shaken at 235 rpm at 37°C until OD_600 _reached approximately 0.8. Cultures were induced with 0.4 mM IPTG and incubated at 30°C for 4 hours. Bacterial cells were harvested by centrifugation and frozen at -20°C. From each expression, 1.0 g of cell paste was lysed in 20 mM Tris-HCl (pH 8.0), 500 mM NaCl, 20% (v/v) Glycerol, Complete-EDTA free protease inhibitor (Roche), 25 units Benzonase, and 0.1 mg/mL lysozyme and sonicated on a Branson Sonifier using a microtip, on 70% duty cycle, output 7, for 45 seconds each. Equal amounts of total protein, as quantified by A_280 _measurement on NanoDrop ND-1000 spectrophotometer were analyzed by SDS-PAGE. Samples were resolved on 4–12% Bis-Tris NuPage 12 well 1 mm gels in 1× MES running buffer, using reducing SDS-PAGE sample buffer and heating samples to 95°C 5 mins prior to loading and visualized by Coomassie blue staining, following manufacturer instructions. Protein bands were visualized on a Kodak Imager and quantified with the software ImageQuant 5.2 (Molecular Devices).

#### *E. coli *expression of pET constructs of Brucella, Burkholderia, Rickettsia targets and pET FtsZ constructs

Pre-cultures (1.2 mL/well, in 96-well deep-well, round bottom block) of 2YT supplemented with antibiotic and 0.5% glucose were inoculated with 15% glycerol stocks made from isolated colonies of BL21(DE3) cells that had been transformed with each sequence verified pET28 construct. Pre-cultures were grown at 37°C, 220 rpm for 16 hours. Cultures (1.2 mL/well, in 96-well deep-well, round bottom block) of 2YT supplemented with antibiotic and Autoinduction System 1 (Novagen) were inoculated with 40 uL of pre-culture and grown for 40 hours at 20°C, 220 rpm. Cultures were harvested by centrifugation and stored at -20°C for at least 1 hour. His-tagged recombinant proteins were purified with magnetic Ni-NTA beads (Qiagen) according to manufacturer protocols on a BioRobot 3000 (Qiagen). Purified recombinant products were analyzed by capillary electrophoresis on Lab Chip 90 (Caliper) using an HT Protein Expression Assay Kit, according to manufacturer protocols.

#### Cell Free Systems

All sequence verified constructs for wheat germ cell free expression (CFS) were purified with an ultra pure plasmid miniprep kit (Marligen) and set to 1 ug/ul. Three expression campaigns were carried out on all FtsZ, NS5B, and P38α constructs. Due to the presence of His tags on all constructs, an H-kit was used. The H-kit contains all components necessary for transcription and translation. All steps were performed according to the manufacturer's instructions for small scale batch mode expression (WEPRO1240H Expression Kit_E_ver. 2.0, Aug. 13, 2007) [42,43]. The RNA was quantified (A_260 _absorbance measurements) and normalized to provide the same amounts of each mRNA sample to the cell free translation mix.

In brief, mRNA encoding each protein was synthesized by *in vitro *transcription (20 ul) of 2 ug the pEU plasmid carrying the gene under control of the SP6 RNA polymerase promoter (37°C 6 hrs). To quantitate RNA, transcription samples were diluted 1:50 and quantitated against a blank of 1:50 dilution of transcription buffer at A_260 _on a Beckman Coulter DU500 spectrophotometer. Thirty-three ug RNA was added to 10.8 ul WEPRO 1240H containing 80 ng/ul creatine kinase and this mixture was laid under 208 ul 1× SUB-AMIX (24 mM HEPES/KOH (pH 7.8), 1.2 mM ATP, 0.25 mM GTP, 16 mM creatine phosphate, 250 units of RNasin (ribonuclease inhibitor), 1 mM dithiothreitol, 0.4 mM spermidine, 0.3 mM each of the 20 amino acids, 2.7 mM magnesium acetate and 100 mM potassium acetate). Translation was carried out overnight at 15–22°C. The following day the bilayer was mixed to homogeneity, and then 100 ul of crude extract was removed, and spun 13,000 rpm 4°C 15 minutes. The supernatant was removed as "soluble" fraction and the small remaining pellet resuspended in 100 ul 10 mM Tris pH 7.5 ("insoluble" fraction). For large scale expression and purification performed by the Desktop II robot (CFS), an H-kit was used, running 6 mls × 6 reaction cups, one construct per cup (six constructs total, NS5B FL, FtsZ FL, and P38α; native and engineered versions of each). The DT II program transcribes, translates, and Ni-IMAC purifies targets with a final elution in 0.5 M imidazole. It was carried out using default parameters (6 hours transcription, 16 hours translation at 15°C) and according to the manufacturer's instructions with the following exception. The robot was paused after the transcription step but before addition of wheat germ to the RNA, so that the RNA for each sample could be manually adjusted to 2600 ug/ml (0.3 ml RNA/sample). After this adjustment, automation was resumed through purification. Protein concentrations of fractions from all were estimated with a detergent compatible Protein Assay (Bio-Rad) according to manufacturer's protocols

### Crystallization

Coordinates and structure factors for a FtsZ double deletant construct (Δ1–11, Δ316–382) with GDP bound have been deposited to the Protein Data Bank with accession code 2RHL. Crystals were obtained at 20°C in Compact Jr. (Emerald BioSystems) sitting drop plates using the CryoF crystallization screen (Emerald BioSystems) and 1 μL of protein with 1 μL of reservoir solution. The following conditions yielded the FtsZ crystals: 30% PEG 200, 100 mM MES pH 6.0, 5% PEG 3000. All crystals were frozen in a fresh drop of their respective crystallant which also served as a cryoprotectant.

#### Data Collection, Structure Solution and Refinement

Data were collected at 100 K at the Advanced Photon Source Structural Biology Center beamline 19BM (Argonne, IL) and were integrated and scaled with HKL2000 [44]. Prior to creating the construct described above, we had crystallized full length *B. subtilis *FtsZ construct (unpublished work). This construct crystallized in the trigonal space group *P*3_2_21 with *a *= 87.1 Å, *c *= 88.1 Å and diffracted to ~ 2.8 Å resolution. The initial structure was solved by molecular replacement with Molrep [45] using data collected at the Advanced Photon Source COMCAT beamline sector 32 and 1FSZ [36] as the search model. The search model contained only homologous residues. All other residues of the search model were replaced with alanine. The structure was built manually with O [46] and refined with Refmac [45]. After successive steps of building and refinement, the *R*-factor converged at ~ 24%. The structure spanned residues Ala 12 to Phe 315 as the N-terminal (1–11) and C-terminal (316–382) residues were disordered. An isomorphous *B. subtilis *FtsZ structure has been deposited to the protein data bank (PDB ID: 2VAM) [47]. The model was improved using Arp/Warp [48] and additional building was carried out with Coot [49]. This refined model was used for molecular replacement searches against the additional data sets. Residues modeled beyond Phe 315 correspond to the rTEV-His-Glu purification tag. All refinements were performed with Refmac. Data collection and refinement statistics are provided in Table 3. Figures were created using the Ribbons software package [50].

**Table 3:** Crystallographic data for *Bacillus subtilis *FtsZ structures.

*Data Collection*	2RHH	2RHJ	2RHL	2RHO
Unit Cell *a*, *b*, *c *(Å)	66.37, 66.37 152.09	74.14, 82.06, 117.51	82.29, 97.58, 135.45	82.30, 97.16, 134.72
Space group	*P*4_1_2_1_2	*C*222_1_	*P*2_1_2_1_2_1_	*P*2_1_2_1_2_1_
Resolution (Å) range/highest resolution shell	50-2.0/(2.07-2.0)	50-1.76/(1.82-1.76)	30-2.45/(2.54-2.45)	50-2.45/(2.54-2.45)
Wavelength (Å)	0.97934	0.97934	0.97934	0.97934
Observed Reflections	267,428	236,328	262,233	228,703
Unique Reflections	23,413	34,949	41,246	40,114
Average I/(sigI)^a^	19.8 (1.4)	27.4 (2.1)	29.0 (2.6)	24.7 (2.0)
*R*_sym _(%)^a,c^	8.7 (44.9)	7.1 (64.2)	6.9 (47.6)	5.0 (56.8)
Completeness (%)^a^	98.3 (88.6)	98.0 (88.8)	99.2 (98.2)	98.1 (94.9)
Redundancy^a^	11.4 (6.0)	6.8 (4.6)	6.4 (5.5)	5.7 (4.7)

*Refinement*				

Resolution (Å)	50 - 2.0	50 – 1.76	30 - 2.45	50 - 2.45
Reflections (working/test)	22,153/1,194	33,173/1,752	38,318/2,039	37,673/2,005
*R*_cryst_/*R*_free _(%)^d^	21.2/25.7	18.0/20.8	22.7/27.0	22.7/27.0
Number of atoms (protein/ligand/water)	2191/5/114	2279/64/172	2199, 2290^b^/ 28/97	2199, 2178^b^/32, 28/99
r.m.s.d. bond lengths (Å)	0.019	0.017	0.015	0.014
r.m.s.d. bond angles (°)	1.580	1.520	1.521	1.508
Average B factor all (Å^2^)	34.2	27.3	67.8	54.8
Average B factor protein (Å^2^)	36.8	25.7	68.0/67.8^b^	55.2/54.7^b^
Average B factor ligand (Å^2^)	31.7	43.6	59.1/53.4^b^	44.4/43.1^b^
Average B factor water (Å^2^)	37.1	39.5	67.7	52.4
Coordinate Error (Å)^e^	0.170	0.104	0.235	0.235

Ramachandran Analysis (%)				

Most Favored	95.8	96.6	94.8/95.4^b^	96.5/96.1^b^
Additionally Allowed	4.2	3.4	5.2/4.6^b^	3.5/3.5^b^

^a^Values in parenthesis are for the highest resolution shells.

^b^Values for chains A and B respectively when applicable.

^c^R_sym _= (Σ_hkl_Σ_i_|I_i_(hkl) - <I(hkl)>|/Σ_hkl_Σ_i _I_i_(hkl), I_i_(hkl) is the intensity measured for the i^th^reflection and <I(hkl)> is the mean intensity of reflections with indices hkl.

^d ^R_cryst _= (Σ_hkl _|| F_obs_(hkl)| - |F_calc_(hkl)||)/Σ_hkl_| F_obs_(hkl)|; R_free _is calculated in an identical manner using 5% of randomly selected reflections.

^e^Estimated standard uncertainty based on *R*_free_.

## Availability and requirements

Gene Composer software can be downloaded from .

## Authors' contributions

AR contributed to experimental strategy, analyzed data, conducted experiments, and drafted the manuscript. DL contributed to experimental strategy, analyzed data, and drafted the manuscript. SL managed all aspects of the protein crystallography and solved the reported structures. JW and MM wrote the Gene Composer database software package. EW and KT cloned the expression constructs. KT and KA purified and analyzed recombinant proteins. AB and LS conceptualized experimental strategy and guided the manuscript preparation. All authors read and approved the final manuscript.

## Supplementary Material

Additional file 1 **Gene and protein sequences for all targets**. Nucleic acid and amino acid sequences for all targets. Click here for file
