# Supplementary material for: Combined protein construct and synthetic gene engineering for heterologous protein expression and crystallization using Gene Composer
Source: BMC Biotechnol. 2009 Apr 21;9:37. doi: 10.1186/1472-6750-9-37 (PMC2680836; doi:10.1186/1472-6750-9-37)
Supplement: Additional file 1 — Gene and protein sequences for all targets. Nucleic acid and amino acid sequences for all targets. [file 1472-6750-9-37-S1.doc]

**Gene and protein sequences for all targets.**

***Bacilus subtilis*: FtsZ**

**Full length:** MLEFETNIDGLASIKVIGVGGGGNNAVNRMIENEVQGVEYIAVNTDAQALNLSKAEVKMQIGAKLTRGLGAGANPEVGKKAAEESKEQIEEALKGADMVFVTAGMGGGTGTGAAPVIAQIAKDLGALTVGVVTRPFTFEGRKRQLQAAGGISAMKEAVDTLIVIPNDRILEIVDKNTPMLEAFREADNVLRQGVQGISDLIATPGLINLDFADVKTIMSNKGSALMGIGIATGENRAAEAAKKAISSPLLEAAIDGAQGVLMNITGGTNLSLYEVQEAADIVASASDQDVNMIFGSVINENLKDEIVVTVIATGFIEQEKDVTKPQRPSLNQSIKTHNQSVPKRDAKREEPQQQNTVSRHTSQPADDTLDIPTFLRNRNKRGENLYFQGHHHHHHEYMPME

Native gene:

atgttggagttcgaaacaaacatagacggcttagcatcaattaaagtaatcggagtaggaggcggcggtaacaacgccgttaaccgaatgattgaaaatgaagtgcaaggagtagagtatatcgcggtaaacacggacgctcaagctcttaacctgtcaaaagcagaagtgaaaatgcaaatcggcgcaaagctgactagaggattgggagcaggtgcgaatccggaagtcgggaaaaaagccgctgaagaaagcaaagagcagattgaagaagcacttaaaggtgctgacatggtattcgtgacagctggtatgggcggcggaacaggaacaggtgccgcaccggttatcgcacaaatcgcgaaagacttaggcgcattaacagtcggcgttgtgacaagaccgtttaccttcgaaggacgcaaaagacagcttcaggctgcaggcggaatctcggcaatgaaagaagcggtggatacactgatcgtgatcccgaacgaccgtatccttgaaattgttgataaaaacacaccgatgcttgaagcattccgcgaagcggataacgtacttcgccaaggggttcaaggtatttctgacttgattgctacacctggtcttatcaaccttgactttgctgatgtgaaaacaatcatgtcaaacaaaggatctgctttgatgggtatcggtattgctactggggaaaatcgcgcggcagaggcagcaaaaaaagcaatttccagcccgcttcttgaagcggccattgacggtgcgcaaggcgtcctcatgaacatcactggaggaacaaacctcagcctatatgaggttcaggaagcagcagacattgtcgcttcggcgtctgatcaagacgtaaacatgattttcggttctgttattaatgaaaatctaaaagatgagattgtggtgacagtgattgcaaccggctttatcgaacaagagaaggacgtgacgaagcctcagcgcccaagcttaaatcaaagcatcaaaacacacaatcaaagtgttccgaagcgtgacgcaaaacgtgaggaacctcagcagcagaacacagtaagccgtcatacttcacagccggctgatgatacgcttgacatcccgacattcttaagaaaccgtaataaacgcggcgaaaacctgtattttcagggccatcatcatcatcatcatgaatatatgccgatggaataatga

Engineered Gene:

atgttggagttcgagaccaacatcgacggtctggcttctatcaaagtgatcggtgttggcggtggcggtaacaacgcggtgaaccgtatgattgagaacgaagtgcaaggcgttgaatacatcgcagtgaacaccgatgcacaagctctgaacctgtccaaggctgaagttaaaatgcaaatcggtgctaaactgacccgtggcctgggcgcgggcgctaacccggaagttggtaagaaggcggcggaggaaagcaaagaacagatcgaagaagccctgaaaggtgctgacatggttttcgttaccgcgggcatgggtggcggtactggcaccggcgctgcacctgttatcgctcaaatcgctaaagatctcggcgctctgactgtcggtgtagttactcgtccttttaccttcgagggtcgtaaacgtcagctgcaggctgccggtggcatctccgctatgaaagaagcggttgacactctgatcgttatcccgaacgaccgtattctggagatcgtagacaagaataccccgatgctggaagcattccgtgaagcagacaacgttctccgtcagggtgttcagggcatctccgaccttatcgccaccccgggtctgatcaacctggacttcgcagacgtaaaaaccatcatgtctaacaaaggctctgctctgatgggcattggtatcgcaactggtgaaaaccgcgctgcagaagcagctaagaaggcgattagctcccctctgctcgaagcggctatcgacggcgcacagggtgttctgatgaacattaccggtggtaccaacctgtccctttacgaagttcaggaagccgctgacatcgtagcgtccgctagcgaccaagatgtgaacatgatctttggctccgttatcaacgaaaaccttaaggacgaaattgtcgtaaccgtaatcgctaccggcttcatcgaacaggagaaggacgttaccaaaccgcagcgtccgtctctgaaccagtccatcaaaacccataaccagtctgttccgaaacgcgacgcgaaacgtgaagagcctcagcaacagaacacggtaagccgtcacaccagccagccggctgacgacactctggatatcccgacgttcctgcgtaaccgtaacaaacgtggtgaaaacctgtattttcagggccatcatcatcatcatcatgaatatatgccgatggaataatgaggatcc

**truncated:**

MASIKVIGVGGGGNNAVNRMIENEVQGVEYIAVNTDAQALNLSKAEVKMQIGAKLTRGLGAGANPEVGKKAAEESKEQIEEALKGADMVFVTAGMGGGTGTGAAPVIAQIAKDLGALTVGVVTRPFTFEGRKRQLQAAGGISAMKEAVDTLIVIPNDRILEIVDKNTPMLEAFREADNVLRQGVQGISDLIATPGLINLDFADVKTIMSNKGSALMGIGIATGENRAAEAAKKAISSPLLEAAIDGAQGVLMNITGGTNLSLYEVQEAADIVASASDQDVNMIFGSVINENLKDEIVVTVIATGFIENLYFQGHHHHHHEYMPME

Native gene:

atggcatcaattaaagtaatcggagtaggaggcggcggtaacaacgccgttaaccgaatgattgaaaatgaagtgcaaggagtagagtatatcgcggtaaacacggacgctcaagctcttaacctgtcaaaagcagaagtgaaaatgcaaatcggcgcaaagctgactagaggattgggagcaggtgcgaatccggaagtcgggaaaaaagccgctgaagaaagcaaagagcagattgaagaagcacttaaaggtgctgacatggtattcgtgacagctggtatgggcggcggaacaggaacaggtgccgcaccggttatcgcacaaatcgcgaaagacttaggcgcattaacagtcggcgttgtgacaagaccgtttaccttcgaaggacgcaaaagacagcttcaggctgcaggcggaatctcggcaatgaaagaagcggtggatacactgatcgtgatcccgaacgaccgtatccttgaaattgttgataaaaacacaccgatgcttgaagcattccgcgaagcggataacgtacttcgccaaggggttcaaggtatttctgacttgattgctacacctggtcttatcaaccttgactttgctgatgtgaaaacaatcatgtcaaacaaaggatctgctttgatgggtatcggtattgctactggggaaaatcgcgcggcagaggcagcaaaaaaagcaatttccagcccgcttcttgaagcggccattgacggtgcgcaaggcgtcctcatgaacatcactggaggaacaaacctcagcctatatgaggttcaggaagcagcagacattgtcgcttcggcgtctgatcaagacgtaaacatgattttcggttctgttattaatgaaaatctaaaagatgagattgtggtgacagtgattgcaaccggctttatagaaaacctgtattttcagggccatcatcatcatcatcatgaatatatgccgatggaataatgaggatcc

Engineered Gene:

atggcttctatcaaagtgatcggtgttggcggtggcggtaacaacgcggtgaaccgtatgattgagaacgaagtgcaaggcgttgaatacatcgcagtgaacaccgatgcacaagctctgaacctgtccaaggctgaagttaaaatgcaaatcggtgctaaactgacccgtggcctgggcgcgggcgctaacccggaagttggtaagaaggcggcggaggaaagcaaagaacagatcgaagaagccctgaaaggtgctgacatggttttcgttaccgcgggcatgggtggcggtactggcaccggcgctgcacctgttatcgctcaaatcgctaaagatctcggcgctctgactgtcggtgtagttactcgtccttttaccttcgagggtcgtaaacgtcagctgcaggctgccggtggcatctccgctatgaaagaagcggttgacactctgatcgttatcccgaacgaccgtattctggagatcgtagacaagaataccccgatgctggaagcattccgtgaagcagacaacgttctccgtcagggtgttcagggcatctccgaccttatcgccaccccgggtctgatcaacctggacttcgcagacgtaaaaaccatcatgtctaacaaaggctctgctctgatgggcattggtatcgcaactggtgaaaaccgcgctgcagaagcagctaagaaggcgattagctcccctctgctcgaagcggctatcgacggcgcacagggtgttctgatgaacattaccggtggtaccaacctgtccctttacgaagttcaggaagccgctgacatcgtagcgtccgctagcgaccaagatgtgaacatgatctttggctccgttatcaacgaaaaccttaaggacgaaattgtcgtaaccgtaatcgctaccggcttcatcgaaaacctgtattttcagggccatcatcatcatcatcatgaatatatgccgatggaataatgaggatcc

**Hepatitis C Virus: NS5B**

**Full length:**

MAHHHHHHSYTWTGALITPCAAEESKLPINALSNSLLRHHNMVYATTSRSAGLRQKKVTFDRLQVLDDHYRDVLKEMKAKASTVKAKLLSVEEACKLTPPHSAKSKFGYGAKDVRNLSSKAVNHIHSVWKDLLEDTVTPIDTTIMAKNEVFCVQPEKGGRKPARLIVFPDLGVRVCEKMALYDVVSTLPQVVMGSSYGFQYSPGQRVEFLVNTWKSKKNPMGFSYDTRCFDSTVTENDIRVEESIYQCCDLAPEARQAIKSLTERLYIGGPLTNSKGQNCGYRRCRASGVLTTSCGNTLTCYLKASAACRAAKLQDCTMLVNGDDLVVICESAGTQEDAASLRVFTEAMTRYSAPPGDPPQPEYDLELITSCSSNVSVAHDASGKRVYYLTRDPTTPLARAAWETARHTPVNSWLGNIIMYAPTLWARMILMTHFFSILLAQEQLEKALDCQIYGACYSIEPLDLPQIIERLHGLSAFSLHSYSPGEINRVASCLRKLGVPPLRVWRHRARSVRARLLSQGGRAATCGKYLFNWAVKTKLKLTPIPAASQLDLSGWFVAGYSGGDIYHSLSRARPRWFMLCLLLLSVGVGIYLLPNR

Native gene:

atggcccatcatcatcatcatcattcctacacatggacaggcgccttgatcacgccatgcgctgcggaggaaagcaagctgcccatcaacgcgttgagcaactctttgctgcgccaccataacatggtttatgccacaacatctcgcagcgcaggcctgcggcagaagaaggtcacctttgacagactgcaagtcctggacgaccactaccgggacgtgctcaaggagatgaaggcgaaggcgtccacagttaaggctaaactcctatccgtagaggaagcctgcaagctgacgcccccacattcggccaaatccaagtttggctatggggcaaaggacgtccggaacctatccagcaaggccgttaaccacatccactccgtgtggaaggacttgctggaagacactgtgacaccaattgacaccaccatcatggcaaaaaatgaggttttctgtgtccaaccagagaaaggaggccgtaagccagcccgccttatcgtattcccagatctgggagtccgtgtatgcgagaagatggccctctatgatgtggtctccacccttcctcaggtcgtgatgggctcctcatacggattccagtactctcctgggcagcgagtcgagttcctggtgaatacctggaaatcaaagaaaaaccccatgggcttttcatatgacactcgctgtttcgactcaacggtcaccgagaacgacatccgtgttgaggagtcaatttaccaatgttgtgacttggcccccgaagccagacaggccataaaatcgctcacagagcggctttatatcgggggtcctctgactaattcaaaagggcagaactgcggttatcgccggtgccgcgcgagcggcgtgctgacgactagctgcggtaacaccctcacatgttacttgaaggcctctgcagcctgtcgagctgcgaagctccaggactgcacgatgctcgtgaacggagacgaccttgtcgttatctgtgaaagcgcgggaacccaagaggacgcggcgagcctacgagtcttcacggaggctatgactaggtactctgccccccccggggacccgccccaaccagaatacgacttggagctgataacatcatgttcctccaatgtgtcggtcgcccacgatgcatcaggcaaaagggtgtactacctcacccgtgatcccaccacccccctcgcacgggctgcgtgggaaacagctagacacactccagttaactcctggctaggcaacattatcatgtatgcgcccactttgtgggcaaggatgattctgatgactcacttcttctccatccttctagcacaggagcaacttgaaaaagccctggactgccagatctacggggcctgttactccattgagccacttgacctacctcagatcattgaacgactccatggccttagcgcattttcactccatagttactctccaggtgagatcaatagggtggcttcatgcctcaggaaacttggggtaccacccttgcgagtctggagacatcgggccaggagcgtccgcgctaggctactgtcccagggggggagggccgccacttgtggcaagtacctcttcaactgggcagtgaagaccaaactcaaactcactccaatcccggctgcgtcccagctggacttgtccggctggttcgttgctggttacagcgggggagacatatatcacagcctgtctcgtgcccgaccccgctggttcatgctgtgcctactcctactttctgtaggggtaggcatctacctgctccccaaccgatagtgaggatcc

Engineered Gene:

atggcccatcatcatcatcatcattcttacacttggaccggcgctctcatcaccccgtgcgcagcagaagaatccaagctgccgattaacgcgctctctaactctcttctgcgccaccacaacatggtttacgcaactacttcccgttctgctggcctgcgtcagaagaaggtaaccttcgaccgtctgcaggtccttgacgaccactaccgtgacgtactgaaagaaatgaaagctaaagcgtccaccgtaaaagctaaactgctgtctgttgaagaagcttgcaaactgaccccgccgcactctgctaagtccaagttcggttacggcgcgaaggacgttcgtaatctgagctccaaggcagttaaccacatccactccgtgtggaaagacctgcttgaggataccgtaaccccgattgacaccaccattatggctaagaatgaagttttctgtgttcagcctgagaaaggtggccgtaagccggctcgtctgattgttttcccggacctgggcgttcgtgtctgcgagaaaatggcgctgtacgacgtggtttctaccctgccgcaagttgtaatgggtagctcttacggcttccaatactccccgggtcagcgcgttgaatttctggtaaacacctggaaatccaaaaaaaatccgatgggtttctcttacgatactcgctgcttcgactccactgttactgagaacgatatccgtgttgaggaatctatctaccagtgctgcgatctggcgccggaagctcgtcaggctatcaaatctctgaccgaacgcctgtatattggcggtccgctgaccaatagcaaaggccagaactgcggctaccgccgttgtcgtgcatccggtgtactgaccacttcctgcggtaacactctgacttgctacctgaaagcatccgctgcttgccgtgcagcaaaactgcaggactgcaccatgctggttaacggcgacgatcttgttgtaatctgcgaatctgctggcacccaggaagacgcggcctccctgcgtgtattcaccgaagctatgacccgttatagcgcaccgccgggcgatccgccgcagccggaatatgatctggaactgatcacttcctgctcctctaacgtttctgttgcccacgatgcttccggcaaacgtgtttactatctgactcgtgacccgaccaccccgctggcgcgtgctgcttgggaaaccgctcgtcacaccccggtaaactcctggctgggtaatatcatcatgtacgctccgaccctgtgggcacgtatgattctgatgactcacttcttctctatcctgctggctcaggaacagctggagaaggcacttgactgtcagatttacggtgcttgctactccattgaaccgctcgacctgccgcagatcatcgaacgtctgcacggtctgagcgctttctccctgcattcctactctccgggcgaaatcaaccgcgttgctagctgcctgcgtaaactgggcgttccgccactgcgcgtttggcgtcaccgtgcacgtagcgtccgcgcacgtctgctgagccagggcggtcgcgcagcgacttgtggtaaatatctgttcaactgggcagttaaaactaaactcaaactgactccgatcccggcggcttctcagctggacctgtctggctggttcgtagcaggttactctggtggtgacatctaccactccctgagccgtgcacgtccgcgttggtttatgctgtgcctgctcctgctcagcgttggtgttggcatctacctgctgccgaaccgttagtgaggatcc

**truncated:**

MAHHHHHHSYTWTGALITPCAAEESKLPINALSNSLLRHHNMVYATTSRSAGLRQKKVTFDRLQVLDDHYRDVLKEMKAKASTVKAKLLSVEEACKLTPPHSAKSKFGYGAKDVRNLSSKAVNHIHSVWKDLLEDTVTPIDTTIMAKNEVFCVQPEKGGRKPARLIVFPDLGVRVCEKMALYDVVSTLPQVVMGSSYGFQYSPGQRVEFLVNTWKSKKNPMGFSYDTRCFDSTVTENDIRVEESIYQCCDLAPEARQAIKSLTERLYIGGPLTNSKGQNCGYRRCRASGVLTTSCGNTLTCYLKASAACRAAKLQDCTMLVNGDDLVVICESAGTQEDAASLRVFTEAMTRYSAPPGDPPQPEYDLELITSCSSNVSVAHDASGKRVYYLTRDPTTPLARAAWETARHTPVNSWLGNIIMYAPTLWARMILMTHFFSILLAQEQLEKALDCQIYGACYSIEPLDLPQIIERLHGLSAFSLHSYSPGEINRVASCLRKLGVPPLRVWRHRARSVRARLLSQGGRAATCGKYLFNWAVKTKLKLTPIPAASQLDLSGWFVAGYSGGDIYHSLSRARPR

Native gene:

atggcccatcatcatcatcatcattcctacacatggacaggcgccttgatcacgccatgcgctgcggaggaaagcaagctgcccatcaacgcgttgagcaactctttgctgcgccaccataacatggtttatgccacaacatctcgcagcgcaggcctgcggcagaagaaggtcacctttgacagactgcaagtcctggacgaccactaccgggacgtgctcaaggagatgaaggcgaaggcgtccacagttaaggctaaactcctatccgtagaggaagcctgcaagctgacgcccccacattcggccaaatccaagtttggctatggggcaaaggacgtccggaacctatccagcaaggccgttaaccacatccactccgtgtggaaggacttgctggaagacactgtgacaccaattgacaccaccatcatggcaaaaaatgaggttttctgtgtccaaccagagaaaggaggccgtaagccagcccgccttatcgtattcccagatctgggagtccgtgtatgcgagaagatggccctctatgatgtggtctccacccttcctcaggtcgtgatgggctcctcatacggattccagtactctcctgggcagcgagtcgagttcctggtgaatacctggaaatcaaagaaaaaccccatgggcttttcatatgacactcgctgtttcgactcaacggtcaccgagaacgacatccgtgttgaggagtcaatttaccaatgttgtgacttggcccccgaagccagacaggccataaaatcgctcacagagcggctttatatcgggggtcctctgactaattcaaaagggcagaactgcggttatcgccggtgccgcgcgagcggcgtgctgacgactagctgcggtaacaccctcacatgttacttgaaggcctctgcagcctgtcgagctgcgaagctccaggactgcacgatgctcgtgaacggagacgaccttgtcgttatctgtgaaagcgcgggaacccaagaggacgcggcgagcctacgagtcttcacggaggctatgactaggtactctgccccccccggggacccgccccaaccagaatacgacttggagctgataacatcatgttcctccaatgtgtcggtcgcccacgatgcatcaggcaaaagggtgtactacctcacccgtgatcccaccacccccctcgcacgggctgcgtgggaaacagctagacacactccagttaactcctggctaggcaacattatcatgtatgcgcccactttgtgggcaaggatgattctgatgactcacttcttctccatccttctagcacaggagcaacttgaaaaagccctggactgccagatctacggggcctgttactccattgagccacttgacctacctcagatcattgaacgactccatggccttagcgcattttcactccatagttactctccaggtgagatcaatagggtggcttcatgcctcaggaaacttggggtaccacccttgcgagtctggagacatcgggccaggagcgtccgcgctaggctactgtcccagggggggagggccgccacttgtggcaagtacctcttcaactgggcagtgaagaccaaactcaaactcactccaatcccggctgcgtcccagctggacttgtccggctggttcgttgctggttacagcgggggagacatatatcacagcctgtctcgtgcccgaccccgctaatgaggatcc

Engineered Gene:

atggcccatcatcatcatcatcattcttacacttggaccggcgctctcatcaccccgtgcgcagcagaagaatccaagctgccgattaacgcgctctctaactctcttctgcgccaccacaacatggtttacgcaactacttcccgttctgctggcctgcgtcagaagaaggtaaccttcgaccgtctgcaggtccttgacgaccactaccgtgacgtactgaaagaaatgaaagctaaagcgtccaccgtaaaagctaaactgctgtctgttgaagaagcttgcaaactgaccccgccgcactctgctaagtccaagttcggttacggcgcgaaggacgttcgtaatctgagctccaaggcagttaaccacatccactccgtgtggaaagacctgcttgaggataccgtaaccccgattgacaccaccattatggctaagaatgaagttttctgtgttcagcctgagaaaggtggccgtaagccggctcgtctgattgttttcccggacctgggcgttcgtgtctgcgagaaaatggcgctgtacgacgtggtttctaccctgccgcaagttgtaatgggtagctcttacggcttccaatactccccgggtcagcgcgttgaatttctggtaaacacctggaaatccaaaaaaaatccgatgggtttctcttacgatactcgctgcttcgactccactgttactgagaacgatatccgtgttgaggaatctatctaccagtgctgcgatctggcgccggaagctcgtcaggctatcaaatctctgaccgaacgcctgtatattggcggtccgctgaccaatagcaaaggccagaactgcggctaccgccgttgtcgtgcatccggtgtactgaccacttcctgcggtaacactctgacttgctacctgaaagcatccgctgcttgccgtgcagcaaaactgcaggactgcaccatgctggttaacggcgacgatcttgttgtaatctgcgaatctgctggcacccaggaagacgcggcctccctgcgtgtattcaccgaagctatgacccgttatagcgcaccgccgggcgatccgccgcagccggaatatgatctggaactgatcacttcctgctcctctaacgtttctgttgcccacgatgcttccggcaaacgtgtttactatctgactcgtgacccgaccaccccgctggcgcgtgctgcttgggaaaccgctcgtcacaccccggtaaactcctggctgggtaatatcatcatgtacgctccgaccctgtgggcacgtatgattctgatgactcacttcttctctatcctgctggctcaggaacagctggagaaggcacttgactgtcagatttacggtgcttgctactccattgaaccgctcgacctgccgcagatcatcgaacgtctgcacggtctgagcgctttctccctgcattcctactctccgggcgaaatcaaccgcgttgctagctgcctgcgtaaactgggcgttccgccactgcgcgtttggcgtcaccgtgcacgtagcgtccgcgcacgtctgctgagccagggcggtcgcgcagcgacttgtggtaaatatctgttcaactgggcagttaaaactaaactcaaactgactccgatcccggcggcttctcagctggacctgtctggctggttcgtagcaggttactctggtggtgacatctaccactccctgagccgtgcacgtccgcgttaatgaggatcc

**Human: P38α**

**Full length:**

MGSSHHHHHHSSGLVPRGSHMSQERPTFYRQELNKTIWEVPERYQNLSPVGSGAYGSVCAAFDTKTGLRVAVKKLSRPFQSIIHAKRTYRELRLLKHMKHENVIGLLDVFTPARSLEEFNDVYLVTHLMGADLNNIVKCQKLTDDHVQFLIYQILRGLKYIHSADIIHRDLKPSNLAVNEDCELKILDFGLARHTDDEMTGYVATRWYRAPEIMLNWMHYNQTVDIWSVGCIMAELLTGRTLFPGTDHIDQLKLILRLVGTPGAELLKKISSESARNYIQSLTQMPKMNFANVFIGANPLAVDLLEKMLVLDSDKRITAAQALAHAYFAQYHDPDDEPVADPYDQSFESRDLLIDEWKSLTYDEVISFVPPPLDQEEMES

Native gene:

atgggcagcagccatcatcatcatcatcacagcagcggcctggtgccgcgcggcagccatatgtctcaggagaggcccacgttctaccggcaggagctgaacaagacaatctgggaggtgcccgagcgttaccagaacctgtctccagtgggctctggcgcctatggctctgtgtgtgctgcttttgacacaaaaacggggttacgtgtggcagtgaagaagctctccagaccatttcagtccatcattcatgcgaaaagaacctacagagaactgcggttacttaaacatatgaaacatgaaaatgtgattggtctgttggacgtttttacacctgcaaggtctctggaggaattcaatgatgtgtatctggtgacccatctcatgggggcagatctgaacaacattgtgaaatgtcagaagcttacagatgaccatgttcagttccttatctaccaaattctccgaggtctaaagtatatacattcagctgacataattcacagggacctaaaacctagtaatctagctgtgaatgaagactgtgagctgaagattctggattttggactggctcggcacacagatgatgaaatgacaggctacgtggccactaggtggtacagggctcctgagatcatgctgaactggatgcattacaaccagacagttgatatttggtcagtgggatgcataatggccgagctgttgactggaagaacattgtttcctggtacagaccatattgatcagttgaagctcattttaagactcgttggaaccccaggggctgagcttttgaagaaaatctcctcagagtctgcaagaaactatattcagtctttgactcagatgccgaagatgaactttgcgaatgtatttattggtgccaatcccctggctgtcgacttgctggagaagatgcttgtattggactcagataagagaattacagcggcccaagcccttgcacatgcctactttgctcagtaccacgatcctgatgatgaaccagtggccgatccttatgatcagtcctttgaaagcagggacctccttatagatgagtggaaaagcctgacctatgatgaagtcatcagctttgtgccaccaccccttgaccaagaagagatggagtcctaatga

Engineered Gene:

atgggcagcagccatcatcatcatcatcacagcagcggcctggtgccgcgcggcagccatatgagccaggaacgtccgaccttctatcgtcaggaactgaacaaaactatctgggaggtgccggaacgttaccagaacctgtccccggttggctctggtgcgtacggttccgtgtgcgcagcttttgacactaaaaccggtctgcgcgtagcggttaagaagctgtctcgtccgttccagtccatcatccatgctaaacgtacttaccgtgagctgcgcctgctgaaacacatgaagcacgaaaacgttatcggtctgctggacgttttcaccccggcacgttccctggaagaattcaacgacgtgtacctggttacccacctgatgggtgcagatctgaacaacattgtgaaatgccagaaactgaccgacgatcacgttcaattcctgatctatcagatcctgcgtggtctgaaatacatccactccgcggacatcatccaccgtgacctgaaaccgtctaacctggcggttaacgaagattgcgaactgaagatcctggatttcggcctggcgcgtcacaccgatgacgaaatgaccggctacgttgcaacccgttggtatcgtgcaccggaaatcatgctgaactggatgcactacaaccagaccgtagatatctggagcgttggttgcatcatggctgaactgctgaccggccgtaccctgtttccgggtaccgaccatattgaccagctgaaactgatcctgcgtctggttggcaccccgggcgctgaactgctgaagaagattagcagcgaatccgcacgtaactacatccaatctctgactcagatgccgaaaatgaacttcgcgaacgttttcatcggcgcgaacccgctggcagttgatctgctggagaagatgctggtgctggactccgacaaacgcatcaccgctgctcaggctctggcacacgcatacttcgcgcaataccacgatccggacgacgagccggtagctgacccgtatgaccaatccttcgaatcccgcgacctgctgatcgacgaatggaaaagcctgacttacgacgaagtgatcagcttcgttccgccgccgctggaccaggaagaaatggaaagctaatga

***Brucella melitensis*, str. biovar Abortus 2308: Lactate/malate dehydrogenase:L-lactate dehydrogenase:TrkA potassium uptake protein [RefSeq YP_415266]; (SSGCID Brab.A.00005.a)**

MGHHHHHHSGEVKPEVKPETHINLKVSDGSSEIFFKIKKTTPLRRLMEAFAKRQGKEMDSLRFLYDGIRIQADQTPEDLDMEDNDIIEAHREQIGGSMARNKIALIGSGMIGGTLAHLAGLKELGDVVLFDIAEGTPQGKGLDIAESSPVDGFDAKFTGANDYAAIEGADVVIVTAGVPRKPGMSRDDLLGINLKVMEQVGAGIKKYAPEAFVICITNPLDAMVWALQKFSGLPAHKVVGMAGVLDSARFRYFLSEEFNVSVEDVTVFVLGGHGDSMVPLARYSTVAGIPLPDLVKMGWTSQDKLDKIIQRTRDGGAEIVGLLKTGSAFYAPAASAIQMAESYLKDKKRVLPVAAQLSGQYGVKDMYVGVPTVIGANGVERIIEIDLDKDEKAQFDKSVASVAGLCEACIGIAPSLK

Native gene:

atgggccaccaccaccaccaccatagcggcgaagtaaaaccggaagtgaagccggagacccacatcaacctgaaggttagcgacggtagcagcgaaatcttcttcaagattaagaagaccaccccgctgcgtcgcctgatggaagcattcgctaaacgccagggcaaggaaatggattccctgcgctttctgtacgacggtatccgtattcaggcagaccagactccggaagatctggacatggaagataacgacattatcgaagcacaccgtgaacaaatcggtggatccatggcacgcaacaagattgccctcatcggctccggcatgatcggcggtacgctcgctcacctggccggtctgaaggaactcggtgacgtcgtccttttcgacattgcggaaggcaccccgcagggcaaggggctggatatcgccgaatcttctccggtcgatggtttcgatgcgaagttcactggcgcgaacgattacgccgccattgaaggcgcagacgtcgtcatcgtcaccgcaggcgtgccgcgcaagccgggcatgagccgcgacgatctcctgggcatcaacctgaaggtgatggaacaggtgggcgcgggcatcaagaaatatgcgccggaagcattcgtcatctgcatcaccaacccgctcgacgccatggtctgggcgctgcagaagttctccggccttccggcccacaaggttgtcggcatggctggcgttctcgacagcgcccgcttccgttatttcctctcggaagaattcaacgtttcggtcgaggacgtcacggtattcgtgctgggtggccacggcgattcgatggttccgctggcgcgctactcgaccgttgccggcattccgctgcctgatctcgtcaagatgggctggaccagccaggacaagctcgacaagatcatccagcgcacccgtgacggcggcgcggaaatcgtgggccttctcaagaccggctcggctttctacgctccggcggcatcggccatccagatggctgaatcctacctcaaggacaagaagcgcgtcctgccggtcgcagcccagctttcgggccagtatggcgtgaaggacatgtatgtgggcgttccgaccgtgatcggtgccaatggcgtggagcgcatcatcgagatcgatctcgacaaggacgagaaggcgcagttcgacaagtcggtggcctccgtcgccggtctgtgcgaagcctgcatcggtattgctccgtcgctgaaataa

Engineered Gene:

atgggccaccaccaccaccaccatagcggcgaagtaaaaccggaagtgaagccggagacccacatcaacctgaaggttagcgacggtagcagcgaaatcttcttcaagattaagaagaccaccccgctgcgtcgcctgatggaagcattcgctaaacgccagggcaaggaaatggattccctgcgctttctgtacgacggtatccgtattcaggcagaccagactccggaagatctggacatggaagataacgacattatcgaagcacaccgtgaacaaatcggtggatccatggctcgtaacaagatcgctctgatcggttccggtatgatcggcggtactctggctcacttagctggcctgaaagaactgggcgacgttgttctgttcgacatcgctgaaggcactccacaaggcaagggcctggatatagctgaatcatctcctgtggacggctttgacgctaaattcactggtgccaacgattacgctgcaatcgaaggtgcagacgttgtgatcgttaccgcaggcgttccacgtaaaccaggtatgtcccgtgacgatctgctgggcatcaacctgaaagttatggaacaggtcggcgccggcattaagaaatacgctccagaagcattcgtcatctgcataaccaaccctctcgacgctatggtttgggcactgcagaaattcagcggtctgccggcacacaaagttgttggcatggcaggtgttctcgactctgctcgtttccgttatttcctctctgaagagttcaacgtaagcgtcgaggacgttactgtgtttgtcctgggtggccacggtgattcgatggtgccgctggctcgttactctaccgttgctggtatcccgctgcctgacttagttaagatgggctggacttcacaggataaactggacaagatcatccagcgtacccgtgacggcggtgcggaaatcgtcggcctgctgaaaactggcagcgctttctacgcaccggctgccagcgccatccagatggctgagtcttatctcaaggacaagaagcgtgtactgccggtcgccgcacagctgagcggtcagtatggtgttaaagatatgtacgttggcgtacctactgttatcggcgcaaacggcgtggagcgtatcatagagatcgatctcgataaggatgagaaagctcagttcgataaatctgtagcatctgtggcaggcctctgtgaagcatgcatcggcatcgctccttccctgaag

***Brucella melitensis*, str. biovar Abortus 2308: CbxX/CfqX superfamily:Disease resistance protein:ATP/GTP-binding site motif A (P-loop):AAA ATPase:AAA ATPase, central region [RefSeq YP_415064]; (SSGCID Brab.A.00013.a)**

MSHHHHHHSGEVKPEVKPETHINLKVSDGSSEIFFKIKKTTPLRRLMEAFAKRQGKEMDSLRFLYDGIRIQADQTPEDLDMEDNDIIEAHREQIGGMSDRNPLIDADRRADEDNTLRPQTLDDFVGQAAARANLKVFIEAAKVRGEALDHVLFVGPPGLGKTTLAQIMAKELGVNFRSTSGPVIAKAGDLAALLTNLEERDVLFIDEIHRLSPAVEEILYPAMEDFQLDLIIGEGPAARSVKIDLAKFTLVAATTRLGLLTTPLRDRFGIPVRLNFYTVEELEYIVRRGARIMQMGISSDGAREVARRSRGTPRIAGRLLRRVRDFALVAGADIIDRRIADEALSRLEVDNRGLDQLDRRYLNIIARNFGGGPVGIETIAAGLSEPRDAIEDIIEPYLIQQGFLQRTPRGRVLTAVAWQHLGLPAPAEIIQQSQYGLFMEDE

Native gene:

atgtcccaccaccaccaccaccatagcggcgaagtaaaaccggaagtgaagccggagacccacatcaacctgaaggttagcgacggtagcagcgaaatcttcttcaagattaagaagaccaccccgctgcgtcgcctgatggaagcattcgctaaacgccagggcaaggaaatggattccctgcgctttctgtacgacggtatccgtattcaggcagaccagactccggaagatctggacatggaagataacgacattatcgaagcacaccgtgaacaaatcggtggcatgagcgaccgcaacccactgatcgacgcagatcgccgtgctgatgaagataataccctccgcccgcagactctggatgacttcgttggccaagctgcagctcgcgcaaacctgaaagtgtttattgaagctgctaaggttcgcggcgaagctctggaccacgtactgttcgtaggcccaccaggtctcggcaagaccactctcgcacaaatcatggctaaggaactcggtgttaacttccgtagcacttctggcccagtgatcgctaaggccggcgatctcgctgctctcctgaccaacctggaagagcgcgacgttctctttatcgacgaaatccaccgcctgagcccagcagttgaagaaatcctgtacccggcaatggaagacttccagctcgacctgatcattggcgaaggtcctgctgctcgttctgttaagattgatcttgctaagtttaccctggtcgcagcaaccactcgtctgggcctgctgaccactcctctccgtgaccgctttggtatccctgttcgcctgaacttctataccgttgaagaactcgaatatatcgttcgccgtggcgcccgcattatgcagatgggcattagctccgacggtgcacgcgaagtagctcgtcgtagccgtggtactcctcgcatcgccggtcgcctgctgcgccgcgtgcgtgatttcgcactggtggcaggtgctgatatcatcgatcgtcgcatcgccgatgaagctctgagccgcctggaagtggataaccgcggcctggatcaactggaccgtcgttatctgaacatcatcgctcgtaacttcggtggcggtccggttggcatcgaaacgattgctgcaggtctgagcgaaccgcgtgacgccatcgaggatatcattgaaccgtacctgatccagcagggcttccttcagcgtactcctcgtggccgtgtgctgaccgctgttgcctggcagcatctcggcctcccggctccggctgagatcatccaacagagccagtatggtctgttcatggaagacgaataa

Engineered Gene:

atgtcccaccaccaccaccaccatagcggcgaagtaaaaccggaagtgaagccggagacccacatcaacctgaaggttagcgacggtagcagcgaaatcttcttcaagattaagaagaccaccccgctgcgtcgcctgatggaagcattcgctaaacgccagggcaaggaaatggattccctgcgctttctgtacgacggtatccgtattcaggcagaccagactccggaagatctggacatggaagataacgacattatcgaagcacaccgtgaacaaatcggtggcatgagcgaccgcaacccactgatcgacgcagatcgccgtgctgatgaagataataccctccgcccgcagactctggatgacttcgttggccaagctgcagctcgcgcaaacctgaaagtgtttattgaagctgctaaggttcgcggcgaagctctggaccacgtactgttcgtaggcccaccaggtctcggcaagaccactctcgcacaaatcatggctaaggaactcggtgttaacttccgtagcacttctggcccagtgatcgctaaggccggcgatctcgctgctctcctgaccaacctggaagagcgcgacgttctctttatcgacgaaatccaccgcctgagcccagcagttgaagaaatcctgtacccggcaatggaagacttccagctcgacctgatcattggcgaaggtcctgctgctcgttctgttaagattgatcttgctaagtttaccctggtcgcagcaaccactcgtctgggcctgctgaccactcctctccgtgaccgctttggtatccctgttcgcctgaacttctataccgttgaagaactcgaatatatcgttcgccgtggcgcccgcattatgcagatgggcattagctccgacggtgcacgcgaagtagctcgtcgtagccgtggtactcctcgcatcgccggtcgcctgctgcgccgcgtgcgtgatttcgcactggtggcaggtgctgatatcatcgatcgtcgcatcgccgatgaagctctgagccgcctggaagtggataaccgcggcctggatcaactggaccgtcgttatctgaacatcatcgctcgtaacttcggtggcggtccggttggcatcgaaacgattgctgcaggtctgagcgaaccgcgtgacgccatcgaggatatcattgaaccgtacctgatccagcagggcttccttcagcgtactcctcgtggccgtgtgctgaccgctgttgcctggcagcatctcggcctcccggctccggctgagatcatccaacagagccagtatggtctgttcatggaagacgaataa

***Brucella melitensis*, str. biovar Abortus 2308: Glyceraldehyde 3-phosphate dehydrogenase:TrkA potassium uptake protein:Glyceraldehyde-3-phosphate dehydrogenase, type I [RefSeq YP_415091]; (SSGCID Brab.A.00052.a)**

MGHHHHHHSGEVKPEVKPETHINLKVSDGSSEIFFKIKKTTPLRRLMEAFAKRQGKEMDSLRFLYDGIRIQADQTPEDLDMEDNDIIEAHREQIGGSMAVRVAINGFGRIGRNILRAIVESGRTDIQVVAINDLGPVETNAHLLRYDSVHGRFPKEVEVAGDTIDVGYGPIKVHAVRNPAELPWKEENVDIALECTGIFTSRDKAALHLEAGAKRVIVSAPADGADLTVVYGVNNDKLTKDHLVISNASCTTNCLAPVAQVLNDTIGIEKGFMTTIHSYTGDQPTLDTMHKDLYRARAAALSMIPTSTGAAKAVGLVLPELKGKLDGVAIRVPTPNVSVVDLTFIAKRETTVEEVNNAIREAANGRLKGILGYTDEKLVSHDFNHDSHSSVFHTDQTKVMDGTMVRILSWYDNEWGFSSRMSDTAVALGKLI

Native gene:

atgggccaccaccaccaccaccatagcggcgaagtaaaaccggaagtgaagccggagacccacatcaacctgaaggttagcgacggtagcagcgaaatcttcttcaagattaagaagaccaccccgctgcgtcgcctgatggaagcattcgctaaacgccagggcaaggaaatggattccctgcgctttctgtacgacggtatccgtattcaggcagaccagactccggaagatctggacatggaagataacgacattatcgaagcacaccgtgaacaaatcggtggatccatggcagttcgcgtcgcaatcaacggttttggccgcattggccgtaacatccttcgcgccatcgtggaatcggggcgcaccgacattcaggtcgtcgccatcaacgatctcggcccggtcgaaaccaacgcacatcttctgcgttatgacagcgttcatggccgtttccccaaggaagtggaggttgcaggcgatacgatcgatgttggctacggcccgatcaaggttcatgccgtccgcaacccggccgaactgccgtggaaggaagaaaacgtcgatatcgcccttgaatgcaccggcattttcacctcgcgcgacaaggcagcacttcatcttgaagctggcgccaagcgcgtcatcgtctccgctcccgcagacggtgccgatctcaccgtcgtctatggtgtcaacaacgacaagctgacgaaggaccatctggtcatctccaacgcttcgtgtaccaccaactgccttgcgccggtggctcaggttctcaacgatactatcggtatcgaaaagggcttcatgaccacgatccactcctatacgggcgaccagccgacgctggacaccatgcacaaggatctctaccgcgcccgcgccgctgccctttccatgatcccgacctcgacgggtgcggccaaggccgtcggtctcgttctgccggaactgaaaggcaagctcgacggcgttgccattcgcgtcccgaccccaaatgtctcggtcgttgatctcaccttcatcgccaagcgtgaaaccaccgttgaagaagtcaacaatgcgatccgcgaagccgccaatggccgcctcaagggcattctcggctataccgatgagaagctcgtctcgcacgacttcaaccacgattcccattcctcggtcttccacaccgaccagaccaaggttatggacggcaccatggtgcgtatcctgtcgtggtacgacaatgaatggggcttctccagccgcatgagcgacaccgccgtcgctttgggcaagctgatctga

Engineered Gene:

atgggccaccaccaccaccaccatagcggcgaagtaaaaccggaagtgaagccggagacccacatcaacctgaaggttagcgacggtagcagcgaaatcttcttcaagattaagaagaccaccccgctgcgtcgcctgatggaagcattcgctaaacgccagggcaaggaaatggattccctgcgctttctgtacgacggtatccgtattcaggcagaccagactccggaagatctggacatggaagataacgacattatcgaagcacaccgtgaacaaatcggtggatccatggcagttcgcgtggccatcaacggtttcggccgcattggccgtaacatccttcgtgcaattgtggaatccggccgtactgatattcaggtagttgctatcaacgacttgggccctgttgaaaccaacgcccatttactccgttacgactccgtacacggccgtttcccgaaagaggttgaagtagctggcgacaccatcgacgtgggttacggtccgatcaaagtccacgcagtgcgtaacccggccgaactgccgtggaaggaggagaatgttgacatagctctggagtgtaccggcatctttactagccgtgataaagctgcactccacctcgaggctggcgctaaacgtgttatagtttccgcaccagcagatggcgctgaccttaccgtggtttacggcgttaacaacgataaactgaccaaagaccacttagtgatctctaacgcttcttgtactacgaactgcctcgcaccggtggcacaggttctgaacgatactatcggtattgagaaaggcttcatgactactatccacagctacaccggtgatcagccgactctcgatacgatgcacaaagatctctaccgcgctcgcgctgcagctctctcgatgatcccaactagcactggtgcagccaaggccgttggtctggtactccctgagctgaagggcaaactcgacggcgttgctatccgcgtgcctactccgaatgtgtccgttgttgacctgacctttattgcaaagcgtgaaaccaccgttgaggaagttaacaacgcgatccgtgaagcagccaacggtcgtctgaaaggtatccttggctacaccgacgagaaactggtatctcacgacttcaaccacgattctcactcttccgtgttccacactgatcaaactaaagttatggatggtactatggttcgcatcctctcctggtacgataatgaatggggtttctcttcccgcatgtctgatactgccgtagccctgggtaagctgatctaa

***Brucella melitensis*, str. biovar Abortus 2308: 3' exoribonuclease:Ribonuclease PH [RefSeq YP_413662]; (SSGCID Brab.A.00060.a)**

MGHHHHHHSGEVKPEVKPETHINLKVSDGSSEIFFKIKKTTPLRRLMEAFAKRQGKEMDSLRFLYDGIRIQADQTPEDLDMEDNDIIEAHREQIGGSMARNKIALIGSGMIGGTLAHLAGLKELGDVVLFDIAEGTPQGKGLDIAESSPVDGFDAKFTGANDYAAIEGADVVIVTAGVPRKPGMSRDDLLGINLKVMEQVGAGIKKYAPEAFVICITNPLDAMVWALQKFSGLPAHKVVGMAGVLDSARFRYFLSEEFNVSVEDVTVFVLGGHGDSMVPLARYSTVAGIPLPDLVKMGWTSQDKLDKIIQRTRDGGAEIVGLLKTGSAFYAPAASAIQMAESYLKDKKRVLPVAAQLSGQYGVKDMYVGVPTVIGANGVERIIEIDLDKDEKAQFDKSVASVAGLCEACIGIAPSLK

Native gene:

atgggccaccaccaccaccaccatagcggcgaagtaaaaccggaagtgaagccggagacccacatcaacctgaaggttagcgacggtagcagcgaaatcttcttcaagattaagaagaccaccccgctgcgtcgcctgatggaagcattcgctaaacgccagggcaaggaaatggattccctgcgctttctgtacgacggtatccgtattcaggcagaccagactccggaagatctggacatggaagataacgacattatcgaagcacaccgtgaacaaatcggtggatccatggcacgcaacaagattgccctcatcggctccggcatgatcggcggtacgctcgctcacctggccggtctgaaggaactcggtgacgtcgtccttttcgacattgcggaaggcaccccgcagggcaaggggctggatatcgccgaatcttctccggtcgatggtttcgatgcgaagttcactggcgcgaacgattacgccgccattgaaggcgcagacgtcgtcatcgtcaccgcaggcgtgccgcgcaagccgggcatgagccgcgacgatctcctgggcatcaacctgaaggtgatggaacaggtgggcgcgggcatcaagaaatatgcgccggaagcattcgtcatctgcatcaccaacccgctcgacgccatggtctgggcgctgcagaagttctccggccttccggcccacaaggttgtcggcatggctggcgttctcgacagcgcccgcttccgttatttcctctcggaagaattcaacgtttcggtcgaggacgtcacggtattcgtgctgggtggccacggcgattcgatggttccgctggcgcgctactcgaccgttgccggcattccgctgcctgatctcgtcaagatgggctggaccagccaggacaagctcgacaagatcatccagcgcacccgtgacggcggcgcggaaatcgtgggccttctcaagaccggctcggctttctacgctccggcggcatcggccatccagatggctgaatcctacctcaaggacaagaagcgcgtcctgccggtcgcagcccagctttcgggccagtatggcgtgaaggacatgtatgtgggcgttccgaccgtgatcggtgccaatggcgtggagcgcatcatcgagatcgatctcgacaaggacgagaaggcgcagttcgacaagtcggtggcctccgtcgccggtctgtgcgaagcctgcatcggtattgctccgtcgctgaaataa

Engineered Gene:

atgggccaccaccaccaccaccatagcggcgaagtaaaaccggaagtgaagccggagacccacatcaacctgaaggttagcgacggtagcagcgaaatcttcttcaagattaagaagaccaccccgctgcgtcgcctgatggaagcattcgctaaacgccagggcaaggaaatggattccctgcgctttctgtacgacggtatccgtattcaggcagaccagactccggaagatctggacatggaagataacgacattatcgaagcacaccgtgaacaaatcggtggatccatggctcgtaacaagatcgctctgatcggttccggtatgatcggcggtactctggctcacttagctggcctgaaagaactgggcgacgttgttctgttcgacatcgctgaaggcactccacaaggcaagggcctggatatagctgaatcatctcctgtggacggctttgacgctaaattcactggtgccaacgattacgctgcaatcgaaggtgcagacgttgtgatcgttaccgcaggcgttccacgtaaaccaggtatgtcccgtgacgatctgctgggcatcaacctgaaagttatggaacaggtcggcgccggcattaagaaatacgctccagaagcattcgtcatctgcataaccaaccctctcgacgctatggtttgggcactgcagaaattcagcggtctgccggcacacaaagttgttggcatggcaggtgttctcgactctgctcgtttccgttatttcctctctgaagagttcaacgtaagcgtcgaggacgttactgtgtttgtcctgggtggccacggtgattcgatggtgccgctggctcgttactctaccgttgctggtatcccgctgcctgacttagttaagatgggctggacttcacaggataaactggacaagatcatccagcgtacccgtgacggcggtgcggaaatcgtcggcctgctgaaaactggcagcgctttctacgcaccggctgccagcgccatccagatggctgagtcttatctcaaggacaagaagcgtgtactgccggtcgccgcacagctgagcggtcagtatggtgttaaagatatgtacgttggcgtacctactgttatcggcgcaaacggcgtggagcgtatcatagagatcgatctcgataaggatgagaaagctcagttcgataaatctgtagcatctgtggcaggcctctgtgaagcatgcatcggcatcgctccttccctgaagtaa

***Burkholderia pseudomallei*, str. 1710b: malate dehydrogenase [RefSeq YP_335954]; (SSGCID Bups.A.00005.a)**

MGHHHHHHSGEVKPEVKPETHINLKVSDGSSEIFFKIKKTTPLRRLMEAFAKRQGKEMDSLRFLYDGIRIQADQTPEDLDMEDNDIIEAHREQIGGSMAKPAKRVAVTGAAGQIAYSLLFRIANGDLLGKDQPVILQLLDLPQAQAAVKGVVMELDDCAFPLLAGVVITDDPKVAFKDADVALLVGARPRSKGMERKDLLSANAEIFTVQGAALNEVASRDVKVLVVGNPANTNAYIAMKSAPDLPKKNFTAMLRLDHNRALSQLAAKSGKPVASIEKLAVWGNHSPTMYPDFRFATAEGESLLKLINDDVWNRDTFIPTVGKRGAAIIEARGLSSAASAANAAIDHVRDWVLGTNGKWVTMGIPSDGSYGIPEDIIYGVPVICENGEYKRVEGLEIDAFSREKMDGTLAELLEERDGVAHLLK

Native gene:

atgggccaccaccaccaccaccatagcggcgaagtaaaaccggaagtgaagccggagacccacatcaacctgaaggttagcgacggtagcagcgaaatcttcttcaagattaagaagaccaccccgctgcgtcgcctgatggaagcattcgctaaacgccagggcaaggaaatggattccctgcgctttctgtacgacggtatccgtattcaggcagaccagactccggaagatctggacatggaagataacgacattatcgaagcacaccgtgaacaaatcggtggatccatggctaagcccgcaaagcgcgttgccgtcaccggtgccgcaggtcagatcgcttactcgctgctgtttcgcatcgcgaacggcgacctgctcggcaaggatcagccggtcatcctgcaactgctcgacctcccgcaagcccaggccgccgtcaaaggcgtcgtgatggaactggacgattgcgcgttcccgctgctcgccggcgtcgtgatcaccgacgacccgaaggtcgcgttcaaggacgccgacgtcgcgctgctggtgggcgcgcgtccgcgctcgaagggcatggagcgcaaggatctgctgtcggcgaacgccgagatcttcacggtccagggcgcggcgctgaacgaagtcgcgagccgcgacgtgaaggtgctggtcgtcggcaacccggcgaacacgaacgcgtacatcgcgatgaagtcggctccggatctgccgaagaagaacttcacggcgatgctgcgcctcgaccacaaccgcgcgctgtcgcagctcgccgcgaagtcgggcaagccggtcgcgtcgatcgagaagctcgccgtgtggggcaaccactcgccgacgatgtaccccgacttccgcttcgcgaccgccgagggcgaatcgctgctgaagctgatcaacgacgacgtgtggaaccgcgacacgttcatcccgactgtcggcaagcgcggcgcggcgatcatcgaagcgcgcggcctgtcgtcggcggcgtcggcggccaacgcggcgatcgaccacgtgcgcgactgggtgctcggcacgaacggcaagtgggtcacgatgggcatcccgtcggacggctcgtacggcatccccgaggacatcatctacggcgtgccggtgatttgcgaaaacggcgagtacaagcgcgtcgagggcctggaaatcgacgcgttctcgcgcgagaagatggacggcacgctcgccgagctgctcgaggagcgcgacggcgtcgcccatctgctgaagtaa

Engineered Gene:

atgggccaccaccaccaccaccatagcggcgaagtaaaaccggaagtgaagccggagacccacatcaacctgaaggttagcgacggtagcagcgaaatcttcttcaagattaagaagaccaccccgctgcgtcgcctgatggaagcattcgctaaacgccagggcaaggaaatggattccctgcgctttctgtacgacggtatccgtattcaggcagaccagactccggaagatctggacatggaagataacgacattatcgaagcacaccgtgaacaaatcggtggatccatggccaaacctgctaagcgcgtggcagttaccggcgccgctggtcagattgcatactctctcctgttccgcatcgccaacggcgacctccttggtaaagatcagccggttattctgcagctgctggatctgccgcaggctcaggcagctgtgaaaggcgttgttatggagctggatgactgcgccttcccgctgctggctggcgtggtgatcacggacgacccgaaggtggcattcaaagacgctgacgtcgcactgctggtgggtgcacgtccacgctccaagggtatggagcgtaaggatctgctgtccgccaacgccgagatctttactgtccagggcgctgcactcaacgaggttgcaagccgtgacgtaaaagttctggtagttggcaaccctgctaacaccaacgcctacatcgctatgaagagcgccccagatctgcctaagaagaattttactgccatgcttcgtctggatcacaaccgtgctctgtctcagctggctgcgaagagcggtaagcctgttgccagcatcgaaaagctcgctgtttggggcaaccactctccaaccatgtacccggacttccgtttcgctactgctgaaggcgaatcgctgcttaagctcatcaacgatgatgtttggaaccgcgacactttcatccctaccgttggtaagcgcggcgctgctatcatcgaagcacgtggcctgtcctctgctgctagcgccgctaatgctgctattgaccacgttcgtgattgggtgctgggcactaacggcaagtgggttaccatgggcatcccgtccgatggttcctacggtatcccggaagatatcatttacggtgtgcctgttatctgcgaaaacggcgaatataagcgtgtagagggcctggaaatcgatgctttctctcgtgaaaaaatggatggcaccctggctgaactgctggaagaacgtgatggcgttgctcatctgcttaaataa

***Burkholderia pseudomallei*, str. 1710b: glutaryl-CoA dehydrogenase [RefSeq YP_334609]; (SSGCID Bups.A.00027.a)**

MGHHHHHHSGEVKPEVKPETHINLKVSDGSSEIFFKIKKTTPLRRLMEAFAKRQGKEMDSLRFLYDGIRIQADQTPEDLDMEDNDIIEAHREQIGGSMAAATFHWDDPLLLDQQLADDERMVRDAAHAYAQGKLAPRVTEAFRHETTDAAIFREMGEIGLLGPTIPEQYGGPGLDYVSYGLIAREVERVDSGYRSMMSVQSSLVMVPIFEFGSDAQKEKYLPKLATGEWIGCFGLTEPNHGSDPGSMVTRARKVPGGYSLSGSKMWITNSPIADVFVVWAKLDEDGRDEIRGFILEKGCKGLSAPAIHGKVGLRASITGEIVLDEAFVPEENILPHVKGLRGPFTCLNSARYGIAWGALGAAESCWHIARQYVLDRKQFGRPLAANQLIQKKLADMQTEITLGLQGVLRLGRMKDEGTAAVEITSIMKRNSCGKALDIARLARDMLGGNGISDEFGVARHLVNLEVVNTYEGTHDIHALILGRAQTGIQAFF

Native gene:

atgggccaccaccaccaccaccatagcggcgaagtaaaaccggaagtgaagccggagacccacatcaacctgaaggttagcgacggtagcagcgaaatcttcttcaagattaagaagaccaccccgctgcgtcgcctgatggaagcattcgctaaacgccagggcaaggaaatggattccctgcgctttctgtacgacggtatccgtattcaggcagaccagactccggaagatctggacatggaagataacgacattatcgaagcacaccgtgaacaaatcggtggatccatggctgccgcaaccttccactgggacgatccgctgctgctcgaccagcagctcgccgacgatgagcgcatggtgcgcgacgccgcgcacgcttacgcgcaaggcaagctcgcgccgcgcgtgaccgaagcgttccgccacgagaccaccgacgcggcgatcttccgtgaaatgggcgagatcggcctcctcggcccgacgatccccgagcagtacggcggccccggcctcgactacgtgagttacgggctcatcgcgcgcgaagtcgagcgcgtcgattcgggttatcggtcgatgatgtcggtgcaatcgtgctcgtgatggtgccgatcttcgaattcggctccgacgcgcagaaggaaaagtacctgccgaagctcgcgacgggcgaatggatcggctgcttcgggctgaccgagccgaaccacggctccgatcccggcagcatggtcacgcgcgcgaggaaggtgccgggcgggtactcgctgtccggctcgaagatgtggatcacgaattcgccgatcgccgacgtgttcgtcgtctgggccaagctcgacgaagacggccgcgacgagattcgcggcttcattctcgaaaagggctgcaaagggctgtcggcgccggcgatccacggcaaggtggggctgcgcgcgtcgatcacgggtgaaatcgtgctcgacgaagcgttcgtccccgaagagaacatcctgccgcacgtgaaggggctgcgcggcccgttcacgtgcctgaactcggcgcgctacggcatcgcgtggggggcgctcggcgcggccgaatcgtgctggcacatcgcgcgccaatatgtgctcgatcgcaagcagttcggccgcccgctcgccgcgaaccagttgatccagaagaagctcgccgacatgcagaccgagatcacgctcggcctgcaaggcgtgctgcggctcggccggatgaaggacgaaggcaccgccgccgtcgagatcacgtcgatcatgaagcgcaattcgtgcggcaaggcgctcgacatcgcccgcctcgcccgcgacatgctgggcggcaatggcatctcggacgaattcggcgtcgcgcgccacctcgtgaacctggaggtggtgaacacgtacgaaggtacgcacgacatccacgcgctgattctcggccgcgcgcagacggggattcaggcgttcttctga

Engineered Gene:

atgggccaccaccaccaccaccatagcggcgaagtaaaaccggaagtgaagccggagacccacatcaacctgaaggttagcgacggtagcagcgaaatcttcttcaagattaagaagaccaccccgctgcgtcgcctgatggaagcattcgctaaacgccagggcaaggaaatggattccctgcgctttctgtacgacggtatccgtattcaggcagaccagactccggaagatctggacatggaagataacgacattatcgaagcacaccgtgaacaaatcggtggatccatggctgcagccaccttccattgggacgatccactgctcctggaccaacagttagctgatgacgaacgtatggttcgcgacgccgcacatgcttatgcccaaggcaaactcgctccgcgtgtgaccgaagcattccgccacgagaccaccgacgccgccatcttccgtgaaatgggcgagattggcctgttaggcccaactatccctgagcagtacggcggccctggtctggattatgtgtcttacggcctgatcgctcgcgaggttgagcgcgttgattctggttatcgttcaatgatgtccgttcagtccagcctcgtcatggtaccgatcttcgagttcggttcggacgcacagaaggagaagtacctgcctaaattagctacgggcgaatggatcggctgctttggtctgaccgaaccaaatcacggctccgacccgggttctatggtcacccgtgcacgtaaagttccgggtggttactctctgagcggttccaagatgtggatcaccaactctcctattgctgatgtgttcgtagtttgggctaaactggacgaagacggtcgtgacgagatccgtggtttcatcctcgagaaaggttgtaaaggtctgtctgctccggcaatccacggcaaagtaggcctgcgtgcatctattactggcgagattgtgcttgacgaagccttcgttccagaagagaacattctgcctcacgtaaagggcctgcgcggtcctttcacttgtctcaatagcgcccgttacggcatcgcctggggcgcattaggcgctgcagaatcttgttggcacattgcccgtcaatatgttctggaccgtaagcagttcggtcgtccgctcgctgcaaaccagctgatccagaaaaaactcgcagatatgcagactgaaatcactctgggcctgcagggtgtgctgcgcctgggccgtatgaaagacgagggtacggcagccgttgaaatcacttccatcatgaaacgtaacagctgtggtaaagctctcgacatcgcccgcctggcccgtgatatgttaggcggcaacggcatctccgacgaatttggtgttgcccgtcacctggtcaaccttgaagtagtaaacacctacgaaggtactcatgacatccacgctctgattctgggtcgtgcccagaccggtatccaggcattcttctaa

***Burkholderia pseudomallei*, str. 1710b: glyceraldehyde-3-phosphate dehydrogenase, type I [RefSeq YP_334837]; (SSGCID Bups.A.00052.a)**

MGHHHHHHSGEVKPEVKPETHINLKVSDGSSEIFFKIKKTTPLRRLMEAFAKRQGKEMDSLRFLYDGIRIQADQTPEDLDMEDNDIIEAHREQIGGSMTIRVAINGYGRIGRNTLRAFYENGKKHDLEIVAINDLGDAKTNAHLTQYDTAHGKFPGEVSVDGDYLVVNGDRIRVLANRNPAELPWGELGVDVVMECTGFFTSKEKASAHLKGGAKKVIISAPGGKDVDATIVYGVNHDVLKAEHTVISNASCTTNCLAPLVKPLNDKIGLETGLMTTIHAYTNDQVLTDVYHEDLRRARSATHSQIPTKTGAAAAVGLVLPELNGKLDGYAIRVPTINVSIVDLSFIAKRDTTAAEVNAIMKEASEGALKGILGYNEAPLVSIDFNHNPASSTFDATLTKVSGRLVKVSSWYDNEWGFSNRMLDTAIALANAK

Native gene:

atgggccaccaccaccaccaccatagcggcgaagtaaaaccggaagtgaagccggagacccacatcaacctgaaggttagcgacggtagcagcgaaatcttcttcaagattaagaagaccaccccgctgcgtcgcctgatggaagcattcgctaaacgccagggcaaggaaatggattccctgcgctttctgtacgacggtatccgtattcaggcagaccagactccggaagatctggacatggaagataacgacattatcgaagcacaccgtgaacaaatcggtggatccatgacgattcgcgttgcaatcaacggttacggccgcatcggccgcaacacgctgcgcgctttctacgaaaacggcaagaagcacgatctcgagatcgttgcgatcaacgatctgggcgacgcgaagaccaacgcgcacctgacgcagtacgacaccgcgcacggcaagttcccgggcgaagtgtcggtcgacggtgattacctcgtcgtgaacggcgacaggattcgcgtgctcgcgaaccgcaacccggccgagctgccgtggggcgagctgggcgtcgacgtcgtgatggaatgcacgggcttcttcacgtcgaaggaaaaggcgagcgcgcacctgaagggcggcgcgaagaaggtgatcatctcggcgccgggcggcaaggacgtcgacgcgacgatcgtctacggcgtgaaccacgacgtgctgaaggccgagcacaccgtcatctcgaacgcatcgtgcacgacgaactgcctggctccgctcgtcaagccgctgaacgacaagatcggcctcgaaaccggcctgatgacgacgattcatgcctacacgaacgaccaggtgctgacggacgtctaccacgaggacctgcgccgcgcgcgctcggcgacgcacagccagatcccgacgaagacgggtgcggcggccgccgtcggcctcgtgctgccggagctgaacggcaagctcgacggctacgcgatccgcgtgccgaccatcaacgtgtcgatcgtcgatctgtcgttcatcgcgaagcgcgacacgaccgcggccgaagtcaacgcgatcatgaaggaagcgtcggaaggcgcgctgaagggcatcctcggctacaacgaggctccgctcgtgtcgatcgacttcaaccacaacccggcttcgtcgacgttcgacgcgacgctcacgaaggtgtcgggccgtctcgtgaaggtgtcgagctggtacgacaacgagtggggcttctcgaaccgcatgctggatacggcgatcgcgctcgcgaacgcgaagtga

Engineered Gene:

atgggccaccaccaccaccaccatagcggcgaagtaaaaccggaagtgaagccggagacccacatcaacctgaaggttagcgacggtagcagcgaaatcttcttcaagattaagaagaccaccccgctgcgtcgcctgatggaagcattcgctaaacgccagggcaaggaaatggattccctgcgctttctgtacgacggtatccgtattcaggcagaccagactccggaagatctggacatggaagataacgacattatcgaagcacaccgtgaacaaatcggtggatccatgactatccgcgtggccattaacggctacggccgcataggccgtaacaccctgcgtgctttctatgagaacggcaagaagcacgacctcgaaatagttgctattaacgacctgggcgacgctaagactaacgcacacctgacccaatacgacaccgcacacggtaaattccctggcgaagtgagcgtcgacggtgattacctggtggttaacggcgatcgtatccgcgtattagctaaccgtaacccagcagaactgccgtggggcgagctgggcgttgatgttgtgatggagtgcaccggtttcttcactagcaaagagaaagcatctgctcacctgaaaggtggtgctaagaaggttattatctctgctccgggcggcaaagatgttgacgcaacgatagtttacggcgttaaccacgatgttctcaaggccgagcacactgttatctctaacgctagctgtactactaactgtcttgcaccactcgttaagccactgaacgacaaaatcggcctcgagaccggcctgatgactactatccacgcttacactaacgaccaggttctgaccgacgtttatcatgaagatctgcgtcgcgcccgttccgcaactcactcccagatcccaactaagacaggtgcagccgcagctgttggtttagtactgcctgaactgaacggcaagctcgatggttacgctatccgtgtaccgaccatcaacgtatcaatcgttgacctgagcttcatcgcaaagcgtgataccaccgcggctgaagttaacgccatcatgaaagaagcatccgaaggtgctctgaaaggcattctgggctacaatgaagctccgcttgtatctatcgacttcaatcacaacccggcctcttccacctttgatgccactctgactaaagtttcaggccgtctcgttaaagtttccagctggtacgataatgagtggggtttctccaaccgtatgctggatacggctatagctctggcaaacgctaagtaa

***Burkholderia pseudomallei*, str. 1710b: beta-lactamase [RefSeq YP_337703]; (SSGCID Bups.A.00104.a)**

MGHHHHHHSGEVKPEVKPETHINLKVSDGSSEIFFKIKKTTPLRRLMEAFAKRQGKEMDSLRFLYDGIRIQADQTPEDLDMEDNDIIEAHREQIGGSMPDSRPFNLPAIRLMNHSPLRRSLLVAAISTPLIGACAPLRGQAKNVAAAERQLRELESTFDGRLGFVALDTATGARIAHRGDERFPFCSTSKMMLCAAVLARSAGEPALLQRRIAYAKGDLIRYSPITEQHVGAGMSVAELCAATLQYSDNTAANLLMALLGGPQAVTAYARSIGDATFRLDRREPELNTALPGDERDTTTPAAMAASVHRLLVGDALGAAQRAQLNAWMLGNKTGDARIRAGVPADWRVADKTGTGDYGTANDIGVAYPPNRAPIVFIVYTTMRNPNAQARDDVIASATRIAARAFA

Native gene:

atgggccaccaccaccaccaccatagcggcgaagtaaaaccggaagtgaagccggagacccacatcaacctgaaggttagcgacggtagcagcgaaatcttcttcaagattaagaagaccaccccgctgcgtcgcctgatggaagcattcgctaaacgccagggcaaggaaatggattccctgcgctttctgtacgacggtatccgtattcaggcagaccagactccggaagatctggacatggaagataacgacattatcgaagcacaccgtgaacaaatcggtggatccatgcccgattcgcgcccgttcaatcttcccgcgatccgcctgatgaatcattctccgttgcgccgctcgctgctcgtcgcagccatttccaccccactgatcggcgcctgcgcgccgctgcgcggccaagcgaaaaacgtcgccgccgccgagcggcaattgcgcgaactcgaatcgacgttcgacggccgcttgggcttcgtcgcgctcgacaccgcgaccggcgcgcgcatcgcgcatcgcggcgacgagcgtttcccgttctgctccacatccaagatgatgctttgcgctgcggtcctcgcgcgcagcgccggcgagcccgcgctgctccagcggcggattgcgtacgcgaagggcgatctcatccgctattcgccgatcaccgagcagcacgtgggcgccggcatgagcgtggccgagctgtgcgcggcgacgctccagtacagcgacaacaccgcggcgaacctgctgatggcgctgctcggcgggccgcaggccgtcaccgcgtatgcgcgctcgatcggcgacgcgacgttccggctcgatcgccgcgagcctgagctgaacacggcgctgcccggcgacgagcgcgatacgacgacgcccgccgcgatggccgcgagcgtgcaccggctgctcgtgggcgacgcgctcggcgccgcgcagcgcgcgcagctcaatgcatggatgctcggcaacaagacgggcgacgcgcgcatccgcgcgggcgtgccggccgactggcgcgtcgccgacaagacgggcacgggcgactacggaacggcgaacgatatcggcgtggcgtatccgccgaatcgcgcgccgatcgtgttcatcgtctatacgacgatgcgcaatccgaacgcacaggcgcgcgacgacgtgatcgcgtcggcgacgcggatcgccgcgcgggcgttcgcctga

Engineered Gene:

atgggccaccaccaccaccaccatagcggcgaagtaaaaccggaagtgaagccggagacccacatcaacctgaaggttagcgacggtagcagcgaaatcttcttcaagattaagaagaccaccccgctgcgtcgcctgatggaagcattcgctaaacgccagggcaaggaaatggattccctgcgctttctgtacgacggtatccgtattcaggcagaccagactccggaagatctggacatggaagataacgacattatcgaagcacaccgtgaacaaatcggtggatccatgccggattcccgtccgttcaatctgccagccatccgcctgatgaaccatagcccgctgcgccgttctctcctggttgccgccatttcaactcctctgatcggtgcttgcgctccgctgcgtggccaggctaagaatgttgcagctgcagaacgtcagctgcgtgagctggagtctaccttcgacggccgtttaggcttcgtggctcttgacaccgctacaggtgctcgcatcgctcaccgcggcgacgaacgtttcccattctgctctacatctaagatgatgctgtgtgcagctgttctggcccgctccgctggcgagcctgctctcctgcaacgtcgtattgcttatgccaaaggcgacctgatccgctatagcccgatcacggaacagcatgttggcgcaggcatgtcggtggctgagctgtgcgcagccactctgcagtactccgacaacactgctgctaacctcctgatggcacttctgggtggccctcaagccgttactgcttacgcccgttctatcggtgatgcaactttccgtctcgaccgtcgtgagccggaactgaacacggcgcttcctggtgacgaacgtgacaccaccactccggccgccatggctgcttccgtccaccgtctgttagttggtgacgccctgggcgcagcacaacgtgctcagctgaacgcctggatgctgggcaacaagactggcgacgctcgtatccgtgctggtgtcccagctgactggcgtgtagctgacaagaccggtaccggtgattacggcactgcaaacgatatcggtgtcgcataccctccgaatcgtgctccaatcgtgtttatagtttacactactatgcgtaacccgaacgctcaggcccgcgacgacgttatcgctagcgccacccgcatagctgcccgtgctttcgcataa

***Rickettsia prowazekii*, str. Madrid E: 2,3,4,5-TETRAHYDROPYRIDINE-2-CARBOXYLATE N-SUCCINYLTRANSFERASE (dapD) [RefSeq NP_220583]; (SSGCID Ripr.A.00002.a)**

MGHHHHHHSGEVKPEVKPETHINLKVSDGSSEIFFKIKKTTPLRRLMEAFAKRQGKEMDSLRFLYDGIRIQADQTPEDLDMEDNDIIEAHREQIGGSMSYIIKEIEEAWQIKENILHDSSKLIKLKKILNESIASLNQGIIRVCEKQGNQWKVNEWVKKAILLYFITTESQLYNNNYNSWYDKVAPKFPADTDKNIFKEAAIRKVPGAIVRTGTYIAKNVVIMPSFINIGAYIDEGTMIDTWATIGSCAQIGKNCHISGGAGIGGVLEPLQAKPVIIEDNCFVGARSEIAEGVIVEEGSVISMGVFIGSSTKIVYRDTGEIIYGRIPAYSVVVPGILPPPEVGKPGLYCVVIVKQVDKTTRGKVSINDLLR

Native gene:

atgggccaccaccaccaccaccatagcggcgaagtaaaaccggaagtgaagccggagacccacatcaacctgaaggttagcgacggtagcagcgaaatcttcttcaagattaagaagaccaccccgctgcgtcgcctgatggaagcattcgctaaacgccagggcaaggaaatggattccctgcgctttctgtacgacggtatccgtattcaggcagaccagactccggaagatctggacatggaagataacgacattatcgaagcacaccgtgaacaaatcggtggatccatgtcctatattattaaagaaattgaagaagcatggcaaattaaggaaaatattcttcatgattcttcaaagctgataaaattaaagaaaatacttaatgagagtatcgcaagtttaaatcaaggtataattcgtgtttgtgaaaagcaaggcaatcagtggaaagttaatgaatgggtaaagaaagctatattgctatattttatcactacagaatcgcagctttataataacaattataatagttggtatgataaagttgctcctaaatttcctgcagatactgataagaatatattcaaagaagcagcgatacgcaaagttccaggagctattgtgagaactggtacttatatcgctaaaaatgttgtaattatgccctctttcattaatatcggtgcttatatagatgagggtacaatgattgatacatgggctactattggatcatgtgctcaaataggtaaaaattgtcatatttccggtggtgcaggaataggtggggtgcttgagccattgcaagccaagcctgttataattgaagataattgctttgttggagcaagatctgaaatagcagaaggtgtaatagtagaggaaggatcagtaattagtatgggtgtgtttattggtagctctacaaaaatagtatatagagatacaggcgaaattatttatggtagaattccagcttattctgttgtggtaccagggatattacctcctcctgaggtaggcaagcctggactttattgcgtagttattgtaaagcaggttgataaaactactcgtggtaaagtaagcatcaatgatttattaaggtga

Engineered Gene:

atgggccaccaccaccaccaccatagcggcgaagtaaaaccggaagtgaagccggagacccacatcaacctgaaggttagcgacggtagcagcgaaatcttcttcaagattaagaagaccaccccgctgcgtcgcctgatggaagcattcgctaaacgccagggcaaggaaatggattccctgcgctttctgtacgacggtatccgtattcaggcagaccagactccggaagatctggacatggaagataacgacattatcgaagcacaccgtgaacaaatcggtggatccatgtcttacatcatcaaagagattgaagaagcatggcagatcaaggagaacatcctgcacgacagcagcaaactgatcaagctgaagaagatcctgaacgagagcatcgcttctctgaaccagggcattatccgcgtttgcgagaagcaaggtaaccagtggaaagttaacgagtgggtcaagaaggctatcttactgtatttcataaccaccgagtctcaactgtataacaataactacaactcgtggtatgacaaggtagctccgaagttcccggctgacacggacaagaacatcttcaaagaggcagcaattcgtaaagttccgggtgcaatagttcgtactggtacctatatcgccaagaatgtagtcattatgccgtcctttatcaacatcggcgcatacattgacgagggcaccatgatagacacttgggctactattggttcgtgtgctcaaatcggtaagaattgtcatatctctggtggtgcaggcatcggtggcgtgctcgagccgctgcaggctaaacctgtgatcatcgaggataactgcttcgttggcgctcgttctgaaatcgctgagggcgtgatcgtagaagagggctctgtaatctccatgggcgtgtttattggctcttctaccaagattgtttatcgtgacaccggcgaaatcatctacggccgtatcccggcttactctgttgtagttccgggcatcctcccgcctccggaagtaggtaaaccaggcttatattgtgtggtgatcgttaaacaggttgacaagactactcgtggcaaagtctccatcaacgacctgctgcgctaa

***Rickettsia prowazekii*, str. Madrid E: Holliday junction DNA helicase RuvB [RefSeq NP_220767]; (SSGCID Ripr.A.00013.a)**

MGHHHHHHSGEVKPEVKPETHINLKVSDGSSEIFFKIKKTTPLRRLMEAFAKRQGKEMDSLRFLYDGIRIQADQTPEDLDMEDNDIIEAHREQIGGSMTNILSPEKSEHDQELPIRPSYLKEFVGQQQIKENLLVFIKAAKSRNEHLDHTLFYGPPGLGKTTLAKIISNEIGGNFKSTAGPAIIKAADLASILTNLEKNDVLFIDEIHRLNTLVEEVLYSAMEDFELDIIIGEGSAARPVKITLPKFTLIGATTRFGLISNPLRDRFGIPMRLNFYNTEELKQVLNRASKLLDIDLTDSGSEEIAKRSRGTPRIALRLLRRIRDFAVVDGKSRIDKEICDFGLKRLTVDSIGLDSNDYRYLKFIADNYHGGPVGIETIAAALSEQRDELEETIEPYLIKIGLVKRTPRGRVITIAAFEHLKMPIPNKSQNQLNILNENE

Native gene:

atgggccaccaccaccaccaccatagcggcgaagtaaaaccggaagtgaagccggagacccacatcaacctgaaggttagcgacggtagcagcgaaatcttcttcaagattaagaagaccaccccgctgcgtcgcctgatggaagcattcgctaaacgccagggcaaggaaatggattccctgcgctttctgtacgacggtatccgtattcaggcagaccagactccggaagatctggacatggaagataacgacattatcgaagcacaccgtgaacaaatcggtggatccatgactaatatattatcacctgaaaaaagtgaacatgatcaagaattgccaataaggccatcatatttgaaagagtttgttggtcaacagcaaattaaagaaaatcttttagtatttatcaaggctgcaaaatctaggaatgagcatcttgatcatactttattttatggcccacctgggcttggtaagactactcttgctaagattatatcaaacgaaatcggtggtaattttaaatctactgctggtcctgctataattaaagcggctgatcttgcttctattcttacaaatcttgaaaaaaatgatgtgttatttattgatgaaatacaccgtctaaataccttggttgaagaagtattatattctgctatggaagattttgagcttgatataattattggtgaaggatctgctgcaaggccagtgaaaataacactaccgaaatttactttaatcggtgctactactcgctttgggctgattagtaatcctttgcgtgataggttcggcattccaatgcgtttaaatttttataatactgaagaattaaaacaagttctaaatagagcaagtaaactacttgacattgatttaacagattctggttctgaagaaatagctaaaagatctcgaggcacaccgagaattgctttaaggttattgcgtcgtataagagattttgcagtagttgatggtaaatcaagaatagataaagaaatttgtgattttgggttaaagcgtttgacagttgatagtataggacttgatagtaatgattatcgttatctaaaattcatagctgataattaccatggcggtcccgtaggaattgaaacaatagcagcagcactttctgaacagcgtgatgaacttgaagaaactatagagccttatcttataaaaataggtttagtaaaaagaactcctagaggtagagtaataacaattgccgcttttgagcatttaaaaatgccaataccgaataaatcacagaatcaattgaatattttaaacgagaatgaatga

Engineered Gene:

atgggccaccaccaccaccaccatagcggcgaagtaaaaccggaagtgaagccggagacccacatcaacctgaaggttagcgacggtagcagcgaaatcttcttcaagattaagaagaccaccccgctgcgtcgcctgatggaagcattcgctaaacgccagggcaaggaaatggattccctgcgctttctgtacgacggtatccgtattcaggcagaccagactccggaagatctggacatggaagataacgacattatcgaagcacaccgtgaacaaatcggtggatccatgactaacatcctgtcccctgagaaatccgaacatgaccaggaactgccgatccgcccgagctacctgaaggaattcgtgggtcagcagcagatcaaagaaaacctcctggtctttatcaaagctgctaaatcccgcaacgaacacctggatcacacgctcttctacggtccacctggtctgggcaaaaccaccctggctaaaatcatctccaacgagatcggcggcaacttcaaatctaccgccggtccggccatcattaaggcagcagacctggcttctatcctcaccaacctggagaaaaacgatgtgctgtttattgacgaaatccatcgcctgaacaccctggtggaagaagttctgtacagcgcaatggaagactttgagctggacatcatcatcggcgagggtagcgctgcccgtccagttaaaatcaccctgccgaaattcaccctgatcggtgctaccactcgtttcggtctgatttccaacccactgcgcgatcgtttcggcatcccgatgcgcctgaacttttacaacaccgaagagctgaaacaggtgctgaatcgtgctagcaagctgcttgacatcgacctgaccgacagcggctccgaagagatcgccaaacgttctcgtggtactccacgtattgctctccgtctgctccgccgcattcgtgatttcgctgtggtggatggtaaatctcgtatcgataaggaaatctgcgacttcggtctgaagcgtctgaccgttgattccatcggcctggactctaacgattaccgctacctgaagttcatcgctgacaactaccacggcggtcctgtgggtattgaaaccatcgcagccgctctgagcgaacaacgcgacgaacttgaagaaaccattgagccttacctgatcaaaatcggtctggttaaacgcaccccgcgcggtcgcgtgatcaccatcgccgcatttgaacacctgaagatgccaatcccgaacaagtcccaaaaccagctgaacatcctgaacgaaaacgaataa

***Rickettsia prowazekii*, str. Madrid E: DNA polymerase III subunit epsilon [RefSeq NP_221085]; (SSGCID Ripr.A.00045.a)**

MGHHHHHHSGEVKPEVKPETHINLKVSDGSSEIFFKIKKTTPLRRLMEAFAKRQGKEMDSLRFLYDGIRIQADQTPEDLDMEDNDIIEAHREQIGGSMSSLREIILDTETTGLDPQQGHRIVEIGAIEMVNKVLTGKHFHFYINPERDMPFEAYKIHGISGEFLKDKPLFKTIANDFLKFIADSTLIIHNAPFDIKFLNHELSLLKRTEIKFLELTNTIDTLVMARNMFPGARYSLDALCKRFKVDNSGRQLHGALKDAALLAEVYVALTGGRQSTFKMINKPDEINNLAVKCVDVQQIKRGIVVKPTKEELQKHKEFIDKILIQA

Native gene:

atgggccaccaccaccaccaccatagcggcgaagtaaaaccggaagtgaagccggagacccacatcaacctgaaggttagcgacggtagcagcgaaatcttcttcaagattaagaagaccaccccgctgcgtcgcctgatggaagcattcgctaaacgccagggcaaggaaatggattccctgcgctttctgtacgacggtatccgtattcaggcagaccagactccggaagatctggacatggaagataacgacattatcgaagcacaccgtgaacaaatcggtggatccatgtcgagtttaagagaaataattttagatactgaaactacgggacttgatccacaacaaggtcatcgaattgttgagatcggtgcaattgagatggtaaataaggtattaacaggcaagcattttcatttttatattaatcctgaacgcgatatgccttttgaggcttataagattcacggtatctcaggagaatttttaaaagataaacctctgtttaagacaatagccaatgattttttaaagtttatagcagatagtacacttattattcataatgctcctttcgatattaaatttctaaatcatgaattatctttattaaagagaaccgaaattaaatttttggaactaacgaatactatagatactctagttatggcaaggaatatgtttcctggagcaagatatagtcttgatgcattatgtaaaagatttaaagttgataattcaggtaggcaacttcatggagctttaaaagatgcagcattacttgcagaagtgtatgttgcactaacaggaggtagacaatctacttttaagatgattaataaacctgatgaaataaacaacttggcagttaagtgtgtagatgtccagcaaataaaaagaggtattgttgttaagcctactaaagaagaattacaaaaacataaagaattcatagataagattttaatacaggcttaa

Engineered Gene:

atgggccaccaccaccaccaccatagcggcgaagtaaaaccggaagtgaagccggagacccacatcaacctgaaggttagcgacggtagcagcgaaatcttcttcaagattaagaagaccaccccgctgcgtcgcctgatggaagcattcgctaaacgccagggcaaggaaatggattccctgcgctttctgtacgacggtatccgtattcaggcagaccagactccggaagatctggacatggaagataacgacattatcgaagcacaccgtgaacaaatcggtggatccatgtcctccctgcgcgaaatcatcctcgatactgaaaccaccggtctggacccgcagcagggtcaccgtatcgtagaaatcggcgctatcgaaatggttaacaaagtactgaccggtaaacattttcacttctacatcaacccggaacgtgacatgccatttgaagcctacaaaattcacggtatttctggcgaatttctgaaggacaaaccgctgttcaaaaccatcgctaacgatttccttaagttcatcgccgactcgaccctcatcatccacaacgcaccgttcgacatcaagttcctgaaccacgagctgagcttgctcaaacgtactgaaattaagttcctggagctgaccaacaccattgataccctggtaatggcccgcaacatgttccctggcgctcgttactccctcgacgctctgtgtaaacgcttcaaagttgataactctggccgtcagctccacggcgcactgaaggacgctgcactcctggcagaggtctatgttgccctgaccggtggccgccagtccactttcaaaatgatcaacaagccagacgaaatcaacaacctggcagtaaaatgcgttgatgttcaacagattaaacgtggcatcgttgttaagcctaccaaagaagaattacagaaacataaagaattcatcgacaaaatcctgatccaggcataa
